# Development of a single-chain fragment variable fused-mutant HALT-1 recombinant immunotoxin against G12V mutated *KRAS c*olorectal cancer cells

**DOI:** 10.7717/peerj.11063

**Published:** 2021-04-15

**Authors:** Michelle Yee Mun Teo, Jeremy Jeack Ceen Ng, Jung Yin Fong, Jung Shan Hwang, Adelene Ai-Lian Song, Renee Lay Hong Lim, Lionel Lian Aun In

**Affiliations:** 1 Department of Biotechnology, Faculty of Applied Sciences, UCSI University, Cheras, Wilayah Persekutuan Kuala Lumpur, Malaysia; 2 Department of Medical Sciences, School of Medical and Life Sciences, Sunway University, Bandar Sunway, Selangor Darul Ehsan, Malaysia; 3 Department of Microbiology, Faculty of Biotechnology and Biomolecular Sciences, Universiti Putra Malaysia, UPM Serdang, Selangor, Malaysia

**Keywords:** KRAS, scfv, Immunotoxin, HALT-1, Hydra, Phagemid, Phage display, Cytotoxic, G12V

## Abstract

**Background:**

*KRAS* oncogenes harboring codon G12 and G13 substitutions are considered gatekeeper mutations which drive oncogenesis in many cancers. To date, there are still no target-specific vaccines or drugs available against this genotype, thus reinforcing the need towards the development of targeted therapies such as immunotoxins.

**Methods:**

This study aims to develop a recombinant anti-m*KRAS* scFv-fused mutant *Hydra* actinoporin-like-toxin-1 (mHALT-1) immunotoxin that is capable of recognizing and eradicating codon-12 mutated k-ras antigen abnormal cells. One G13D peptide mimotope (164-D) and one G12V peptide mimotope (68-V) were designed to elicit antigen specific IgG titres against mutated K-ras antigens in immunised Balb/c mice. The RNA was extracted from splenocytes following ELISA confirmation on post-immunized mice sera and was reverse transcribed into cDNA. The scFv combinatorial library was constructed from cDNA repertoire of variable regions of heavy chain (V_H_) and light chain (V_L_) fusions connected by a flexible glycine-serine linker, using splicing by overlap extension PCR (SOE-PCR). Anti-m*KRAS* G12V and G13D scFvs were cloned in pCANTAB5E phagemid and superinfected with helper phage. After few rounds of bio-panning, a specific m*KRAS* G12V and G13D scFv antibody against G12V and G13D control mimotope was identified and confirmed using ELISA without any cross-reactivity with other mimotopes or controls. Subsequently, the anti-m*KRAS* scFv was fused to mHALT-1 using SOE-PCR and cloned in pET22b vector. Expressed recombinant immunotoxins were analyzed for their effects on cell proliferation by the MTT assay and targeted specificity by cell-based ELISA on *KRAS*-positive and *KRAS*-negative cancer cells.

**Results:**

The V_H_ and V_L_ genes from spleen RNA of mice immunized with 164-D and 68-V were amplified and randomly linked together, using SOE-PCR producing band sizes about 750 bp. Anti-m*KRAS* G12V and G13D scFvs were constructed in phagemid pCANTAB5E vectors with a library containing 3.4 × 10^6^ and 2.9 × 10^6^ individual clones, respectively. After three rounds of bio-panning, the anti-m*KRAS* G12V-34 scFv antibody against G12V control mimotope was identified and confirmed without any cross-reactivity with other controls using ELISA. Anti-m*KRAS* G12V-34 scFv fragment was fused to mHALT-1 toxin and cloned in pET22b vector with expression as inclusion bodies in *E. coli* BL21(DE3) (molecular weight of ~46.8 kDa). After successful solubilization and refolding, the mHALT-1-scFv immunotoxin exhibited cytotoxic effects on SW-480 colorectal cancer cells with IC_50_ of 25.39 μg/mL, with minimal cytotoxicity effect on NHDF cells.

**Discussion:**

These results suggested that the development of such immunotoxins is potentially useful as an immunotherapeutic application against *KRAS*-positive malignancies.

## Introduction

Colorectal cancer continues to rise as one of the most frequent causes of cancer-related deaths in both developing and developed countries, especially amongst east Asian nations. While the use of immunotherapies such as anti-epidermal growth factor receptor (EGFR) monoclonal antibodies have found reasonable ground against EGFR positive colorectal cancer patients, the absence of effective targeted immunotherapies against EGFR positive patients harboring a mutated Kirsten rat sarcoma viral oncogene homolog (*KRAS*) gene have yet to be addressed. This leaves mutated *KRAS* patients with non-specific anti-neoplastic drugs as their sole treatment option with a statistically poor 5-year prognosis and a rapid deterioration in the quality of life (National Cancer Registry, National Cancer Institute, Ministry of Health Malaysia, 2018). Past efforts to inhibit mutated KRAS proteins either directly or indirectly have been attempted, but met with little success. Examples of such targets and biologics include farnesyltransferase inhibitors to block KRAS membrane localization, PDEδ, and other effector signaling pathways downstream of KRAS (Ryan & Corcoran, 2018).

Oncogenic *KRAS* mutations are commonly found in colorectal cancers (40–50%), making it the most prominently mutated proto-oncogenes known to date (Jancik et al., 2010). Of those, about 83% involve codon 12% and 14% involve codon 13, whereas codon 61 accounts for a minor proportion (2%) (Hobbs, Der & Rossman, 2016). Therefore, there is an ongoing dire need to improve on targeted cancer therapies for the management of *KRAS*-positive colorectal cancer cases.

An approach using third generation recombinant immunotoxins, which are toxin moieties fused to the single-chain variable fragment (scFv) portion of antibodies, have gained much attraction as cancer therapeutics following their ability to specifically target and eradicate cancer cells. The use of scFvs in recombinant immunotoxins provides several advantages in therapeutic applications due to reduced immunogenicity, rapid blood clearance, lower retention times and better tissue penetration (Ahmad et al., 2012). A comprehensive list of past scFv based immuno-therapeutics tested in both pre-clinical and clinical trial settings was discussed and summarized (Fercher et al., 2018).

Through a phage display scFv library established in this study, sequences capable of binding mutated K-ras antigens were panned out and fused with a mutant *Hydra* actinoporin-like-toxin-1 (mHALT-1) from *Hydra magnipapillata*. This small 20.8 kDa pore forming toxin is capable of binding preferentially to the sphingomyelin-associated membrane, and form cation-selective pores which lyses targeted cells due to an imbalance in osmotic pressure (Liew et al., 2015). It acts by forming pores in the cells’ lipid membrane which leads to osmotic swelling and ultimately, cell death (Tejuca, Anderluh & Serra, 2009). Such *Hydra* toxins are extremely potent and can be exploited as cell-directed immunotoxins. Through site-directed mutagenesis, the toxin’s affinity towards sphingomyelin was removed, enabling the toxin to specifically target cells solely through the scFv moiety, thus conferring targeted specificity (Liew et al., 2015). Since cell death caused by mHALT-1 does not require cellular internalization to reach a cytosolic target, mHALT-1 works immediately upon contact with the lipid cell membrane, hence, becoming an ideal toxin moiety candidate for immunotoxins.

Currently, several recombinant immunotoxins are undergoing clinical trials against different types of cancers, however, none is currently in the pipeline against *KRAS*-positive cancers. Therefore, this study reports a proof-of concept approach employing a 3rd generation anti-m*KRAS* scFv immunotoxin potentially capable of target-specific eradication of *KRAS*-positive colorectal cancer cells.

## Materials & Methods

### Construction of phage-displayed scFv library

Total RNA was extracted from homogenized mice splenocytes immunized with 164-D (YKLVVVGAGDVYKSA) and 68-V (YKLDVVGAVGVGKSA) mimotopes (Ng et al., 2018) using RNeasy Mini kit (Qiagen, Germantown, MD, USA) according to the manufacturer’s instructions. Reverse-transcription with the RNA extract was performed with Tetro cDNA synthesis kit (Bioline, London, United Kingdom) following the manufacturer’s protocol. Primers for PCR amplification of scFv construction were designed according to Table 1. In the first round of PCR, V_H_ and V_L_ were amplified separately by PCR reactions containing eight forward primers with four reverse primers adapted from Zhou, Fisher & Papas (1994) with modifications. PCR products were then purified using a gel extraction kit (iNtRON, Korea). The assembled scFvs were double digested with *Sfi*I and *Not*I (NEB, Hitchin, UK) and ligated into pCANTAB5E phagemid vector (a molar vector to insert ratio of 1:3), using electroligase (NEB, Hitchin, UK). The resulting ligation products were electroporated into *E. coli* TG1 at 1.8 kV, 5 ms. The library was then grown in 1 mL 2x Bacto tryptone (YT)-G medium (2% (v/v) glucose) for 1 h and then plated on SOBAG plates containing 100 μg/mL ampicillin and 2% glucose. Colonies were scraped off into 2x YT medium and pooled to make library glycerol stocks for storage at −80 °C. The glycerol stocks of scFv libraries were grown in 2x YT-G media (2% (v/v) glucose) and incubated at 37 °C with shaking until it reached mid-log phase (OD_600_: 0.5). A final concentration of 100 μg/mL ampicillin and 4 × 10^10^ pfu of M13K07 helper phage were added to the culture and grown at 37 °C for 1 h with shaking. The phage-infected *E. coli* were pelleted by centrifugation and incubated overnight with 2x YT-AK media (100 μg/mL ampicillin and 50 μg/mL kanamycin). Rescued phage particles in the supernatant were precipitated by adding 1/5 volume of 20% 2.5 M PEG/NaCl and incubated on ice for 1 h. The phage pellets were collected by centrifugation at 10,000×*g* for 20 min at 4 °C and resuspended in 1/50 volume of PBS. Following centrifugation at 12,000×*g* for 15 min at 4 °C, the supernatant was filtered through 0.45 μm filter to remove bacterial cells.

**Table 1:** PCR primers for the amplification of murine V_H_, V_L_ and scFv fragments.

Primer	Oligonucleotide sequence (5′–3′)
VH.FOR1 VH.FOR2 VH.FOR3 VH.FOR4 VH.FOR5 VH.FOR6 VH.FOR7 VH.FOR8 VH.REV1 VH.REV2 VH.REV3 VH.REV4	*Primers for the variable region of murine heavy chain* *GC***GGCCCAGCCGGCC**ATGGCCGATGTGAAGCTTCAGGAGTC GC**GGCCCAGCCGGCC**ATGGCCCAGGTGCAGCTGAAGGAGTC GC**GGCCCAGCCGGCC**ATGGCCCAGGTGCAGCTGAAGCAGTC GC**GGCCCAGCCGGCC**ATGGCCCAGGTTACTCTGAAAGAGTC GC**GGCCCAGCCGGCC**ATGGCCGAGGTCCAGCTGCAGCAGTC GC**GGCCCAGCCGGCC**ATGGCCCAGGTCCAACTGCAGCAGCC GC**GGCCCAGCCGGCC**ATGGCCGAGGTGAAGCTGGTGGAGTC GC**GGCCCAGCCGGCC**ATGGCCGAGGTGAAGCTGGTGGAATC cgatccgccaccgccagagccacctccgcctgaaccgcctccaccTGCAGAGACAGTGACCAGAGT cgatccgccaccgccagagccacctccgcctgaaccgcctccaccTGAGGAGACTGTGAGAGTGGT cgatccgccaccgccagagccacctccgcctgaaccgcctccaccTGAGGAGACGGTGACTGAGGT cgatccgccaccgccagagccacctccgcctgaaccgcctccaccTGAGGAGACGGTGACCGTGGT
VL.FOR1 VL.FOR2 VL.FOR3 VL.FOR4 VL.FOR5 VL.FOR6 VL.FOR7 VL.FOR8 VL.REV1 VL.REV2 VL.REV3 VL.REV4	*Primers for the variable region of the murine light chain* ggtggaggcggttcaggcggaggtggctctggcggtggcggatcgGATGTTTTGATGACCCAAACT ggtggaggcggttcaggcggaggtggctctggcggtggcggatcgGATATTGTGATGACGCAGGC ggtggaggcggttcaggcggaggtggctctggcggtggcggatcgGACATTGTGCTGACCCAATCT ggtggaggcggttcaggcggaggtggctctggcggtggcggatcgGACATTGTGATGACCCAGTCT ggtggaggcggttcaggcggaggtggctctggcggtggcggatcgGATATTGTGCTAACTCAGTC ggtggaggcggttcaggcggaggtggctctggcggtggcggatcgGATATCCAGATGACACAGAC ggtggaggcggttcaggcggaggtggctctggcggtggcggatcgGACATCCAGCTGACTCAGTC ggtggaggcggttcaggcggaggtggctctggcggtggcggatcgCAAATTGTTCTCACCCAGTCT CTGCGGCCGCCCGTTTCAGCTCCAGCTTG CTGCGGCCGCCCGTTTTATTTCCAGCTTGGT CTGCGGCCGCCCGTTTTATTTCCAACTTTG CTGCGGCCGCGGATACAGTTGGTGCAGCATC

**Note:**

FOR, forward; REV, reverse; Bold letters, *Sfi*I restriction site; Underlined letters, *Not*I restriction site; Lower case letters, peptide linker sequence to generate a peptide linker fragment for V_H_ and V_L_ PCR assembly.

### Antigen-specific phage selection by biopanning

The PEG-precipitated phage library was subjected to three rounds of selections. Panning selection of the phage was carried out on 96-well immunoplate (SPL, South Korea) immobilized with G12V and G13D control peptide antigens, using 50 μg for the first round, 25 μg for the second round, and 15 μg for the third round diluted in 100 μL of PBS overnight at 4 °C. Peptide-coated wells were blocked with 2% (w/v) skim milk powder in PBS at 37 °C for 2 h. The pre-blocked phage-scFv was added and incubated at 37 °C for 2 h. After washing with 0.05% (v/v) of Tween-20 in PBS (PBST), 100 µL of log-phase *E. coli* TG1 cells were added and incubated at 37 °C for 30 min with shaking. An aliquot of the dilution was plated on 2x YT-AG (100 μg/mL ampicillin and 2% (v/v) glucose) plates and incubated at 30 °C overnight.

### Rescue and screening of phage antibodies by phage-ELISA

Individual phagemid clones were cultured in cluster tubes (Corning, New York, NY, USA) with 400 μL of 2x YT-AG (100 μg/mL ampicillin and 2% (v/v) glucose) media and incubated at 30 °C overnight with shaking at 250 rpm. The recombinant phages were rescued by transferring 40 μL overnight culture to 400 μL of 2x YT-AG containing 2.5 × 10^10^ pfu of M13K07. Following incubation for 2 h at 37 °C with shaking at 150 rpm, the cells were pelleted at 1,500×*g* and resuspended in 400 μL of 2x YT-AK (100 μg/mL ampicillin and 50 μg/mL kanamycin) media. The culture was incubated overnight at 37 °C and the phage-antibody supernatant was used to perform phage-ELISA. MaxiBinding microtiter plates (SPL, South Korea) were coated with 10 μg/mL control peptide antigens (100 μL) diluted in PBS, pH 7 at 4 °C overnight. The coated plates were washed two times with PBS and blocked with 1% (w/v) bovine serum albumin (BSA) for 2 h at 37 °C. A total of 100 μL of pre-blocked phage antibody supernatant (80 μL + 20 μL 1% (w/v) BSA) was added and incubated for 2 h at 37 °C. Unbound antibodies were removed by washing three times with 0.05% (v/v) PBST and PBS, respectively. After the washings, 100 μL of diluted horseradish peroxidase (HRP)-conjugated anti-M13 antibody (Abcam, Cambridge, England, UK) in 1% (w/v) BSA (1:5,000) was added, and the plates were incubated for 1 h at 37 °C. The wells were washed as described above. Hundred microliters of 3,3′,5,5′-Tetramethylbenzidine (TMB) substrate was added and the reaction was stopped with 50 μL of 1N sulphuric acid (H_2_SO_4_) per well. OD_450_ was determined using FLUOstar^®^ Omega (BMG Labtech, Germany). Positive responses were determined with OD_450_ readings, using a threshold of at least two times that of the control (1% BSA) (Singh et al., 2010). BSA-coated wells consist of phage antibodies without antigen coating, while wt M13K07 helper phage controls consist of antigen-coated wells with the addition of wt helper phage. wt M13K07 helper phage controls were used to identify background signals. All samples and controls were assayed in three replicates, and all data were expressed as mean values ± standard deviation indicated by error bars.

### scFv DNA sequencing analysis

Positive clone plasmids were isolated with iNtRON Biotechnology DNA-spin^™^ plasmid DNA purification kit according to the manufacturer’s protocol. The scFv DNA region was sequenced using pCANTAB5E sequencing forward primer R1 5′-CCATGATTACGCCAAGCTTTGGAGCC-3′. Sequencing results were aligned using the Clustal Omega Multiple Alignment from BioEdit version 7.1.9 (Tom Hall, Fort Worth, TX, USA). The full-length V_H_ and V_L_ chain region sequences were numbered according to the sequence analysis tool IgBLAST (Ye et al., 2013) for the determination of complementarity-determining region (CDR) and the germline origins of V regions of scFv clones.

### Expression of soluble scFv antibodies

Positive E-tag fusion scFv clones were transformed into non-suppressor *E. coli* HB2151 cells. The recombinant anti-m*KRAS* E-tag proteins were expressed and localized to the periplasmic space of E. *coli*. Briefly, 400 μL of log phase *E. coli* HB2151 cells were infected with 2 μL of recombinant phage antibodies and incubated at 37 °C for 30 min with shaking at 150 rpm. To ensure resulting colonies were true nal^r^ (nalidixic acid resistant) and not carried over from infected *E. coli* TG1 cells, the culture was streaked on SOBAG-N plate (SOBAG containing 100 μg/mL nalidixic acid). Following overnight incubation at 30 °C, a single colony of infected *E. coli* HB2151 was picked and inoculated in five mL of 2x YT-AG media (100 μg/mL ampicillin and 2% (v/v) glucose) at 30 °C overnight. The overnight culture (five mL) was inoculated in 50 mL fresh 2x YT-AG (100 μg/mL ampicillin and 2% (v/v) glucose) and incubated at 30 °C for 1 h with shaking at 250 rpm. Following centrifugation at 1,500×*g* for 20 min, the pellet was resuspended in 50 mL of 2x YT-AI media (100 μg/mL ampicillin and 1 mM IPTG), and incubated at 30 °C with shaking at 250 rpm. After 4 h of incubation, the culture was divided into two separate 50 mL tubes and centrifuged as detailed above. The pellets were treated with {[2-hydroxy-1,1-bis(hydroxymethyl)ethyl]amino} ethanesulfonic acid (TES) buffer by mild osmotic shock to liberate the recombinant anti-m*KRAS* scFv antibodies from the periplasmic space of host *E. coli* HB2151 cells. Cell pellets were resuspended in 0.5 mL of ice-cold 1X TES after which 0.75 mL of ice-cold 1/5 TES buffer was added, and incubated on ice for 30 min. Following centrifugation at 13,000×*g* for 10 min, the supernatant containing the soluble periplasmic fraction was used for monoclonal-ELISA. The supernatant was analyzed by SDS-PAGE and western blotting with anti-E tag HRP-conjugated antibodies.

### Monoclonal-ELISA

The antigen specificity of soluble scFv antibody was determined by monoclonal-ELISA using an anti-E-tag antibody. MaxiBinding microtiter plates were coated with 10 μg/mL wildtype or control peptide antigens (G12V, G13D) (100 μL) diluted in PBS at 4 °C and blocked with 1% (w/v) BSA for 2 h at 37 °C. The wells were washed three times with PBS and 100 μL of pre-blocked soluble scFv antibodies (80 μL + 20 μL 1% (w/v) BSA) was added. Plates were incubated for 2 h at 37 °C. The wells were washed three times with 0.05% (v/v) PBST and PBS, respectively. HRP-conjugated anti-E tag antibody (Abcam, Cambridge, England, UK) diluted in 1% (w/v) BSA (1:5,000) (100 μL) was added, and the plates were incubated for 1 h at 37 °C. The wells were washed as described above. 100 μL of TMB substrate was added and the reaction was stopped by adding 50 μL of 1M H_2_SO_4_ per well. The absorbance at 450 nm was measured using FLUOstar^®^ Omega (BMG Labtech, Germany). Positive responses were identified with OD_450_ readings using a threshold of at least two times that of the control (1% BSA) (Della Cristina et al., 2015). Non-coated wells with sera and blank with antigen-coated wells without sera controls were used to identify background signals. All samples and controls were expressed as mean absorbance values ± standard deviation by subtracting the mean absorbance for non-coated wells with sera and wells without the sera, assuming each antigen was coated consistently between plates. Differences between test and control groups were analyzed using paired Student’s *t*-test. Significance was set at *p* < 0.05 (*) and *p* < 0.01 (**) for all tests.

### Construction of scFv-eGFP

The anti-m*KRAS* G12V-34 scFv and enhanced green fluorescent protein (eGFP) were amplified separately and were purified using the gel extraction kit (iNtRON, Korea). The PCR products were assembled via SOE-PCR and double digested with *Xba*I and HindIII (NEB, Hitchin, UK). The digested PCR products were ligated into pET-28a expression vector (a molar vector to insert ratio of 1:3), using electroligase (NEB, Hitchin, UK). The ligation products were electroporated into *E.coli* BL21(DE3) at 1.8 kV, 5 ms. One milliliter of pre-warmed SOC media was added to the electroporated cells, which were then incubated at 37 °C for 1 h with shaking at 150 rpm. The recovered cells were plated out into LB-kan plates and incubated at 37 °C overnight. Positive clones were isolated with iNtRON Biotechnology DNA-spin^™^ plasmid DNA purification kit according to the manufacturer’s protocol. The scFv-eGFP DNA region was sequenced using pET22b sequencing forward primer 5′-TAATACGACTCACTATAGGG-3′. Positive scFv-eGFP clones were expressed in *E. coli* BL21(DE3) in the presence of 1 mM IPTG for 4 h at 37 °C with shaking at 200 rpm. The cells were harvested by centrifugation at 7,000×*g* for 10 min and resuspended in 20 mM Tris-CI buffer (pH 8.0) with the addition of 1 mM phenylmethylsulfonyl fluoride (PMSF) prior to sonication at 130 watt and 20 kHz for a total of 10 min on ice.

### Protein expression, isolation, affinity purification and refolding of scFv-eGFP

His-tag fusion scFv-eGFP was expressed in *E. coli* BL21(DE3) in the presence of 1 mM IPTG for 3 h at 30 °C with shaking at 150 rpm. The cells were harvested by centrifugation at 7,000×*g* for 10 min and resuspended in 20 mM Tris-CI buffer (pH 8.0) with the addition of 1 mM phenylmethylsulfonyl fluoride (PMSF) prior to sonication at 130 watt and 20 kHz for a total of 10 min on ice. The inclusion bodies were resuspended in inclusion body washing buffer (2 M urea; 20 mM Tris-CI; 0.5 M NaCI; 2% Triton X-100; pH 8.0) and solubilized in solubilization buffer (8 M urea; 20 mM sodium phosphate; 50 mM 2-mercaptoethanol; pH 7.8) overnight at RT. The solubilized inclusion bodies were further purified with nickel-nitrilotriacetic acid (Ni-NTA) resin (Qiagen, Hilden, Germany) by using a pH gradient (pH 4.5–7.8). The purified His-tag fusion scFv-eGFP were refolded by stepwise dialysis with phosphate refolding buffer containing L-arginine with decreasing urea concentrations (6 M, 4 M, 2 M and 1 M). Refolded proteins were filtered with a 0.22 µm filter and measured by using Bradford assay (Bio-rad, Irvine, CA, USA) with varying concentrations of BSA as a standard.

### Construction of recombinant immunotoxin

Primers for PCR amplification of anti-m*KRAS* G12V-34 scFv and mHALT-1 were amplified separately using primers sets listed in Table 2. A total of two recombinant immunotoxins with opposing orientations were constructed using SOE-PCR, (1) scFv-mHALT-1; N-terminal anti-m*KRAS* scFv and C-terminal mHALT-1, and (2) mHALT-1-scFv; N-terminal mHALT-1 and C-terminal anti-m*KRAS* scFv. Both fragments were amplified separately by PCR. The PCR products were then purified using a gel extraction kit (iNtRON, Korea). For SOE-PCR, purified anti-m*KRAS* G12V-34 scFv and mHALT-1 amplicons were used. The assembled recombinant immunotoxins were double digested with *Xho*I and *Nco*I (NEB, Hitchin, UK) and ligated into pET22b expression vector (a molar vector to insert ratio of 1:3), using electroligase (NEB, Hitchin, UK). The resulting ligation products were electroporated into *E. coli* BL21(DE3) at 1.8 kV, 5 ms. One milliliter of pre-warmed SOC media was added to the electroporated cells, which were then incubated at 37 °C for 1 h with shaking at 200 rpm. The recovered cells were plated out into LB-amp plates and incubated at 37 °C overnight. All positive clone plasmids were isolated with iNtRON Biotechnology DNA-spin^™^ plasmid DNA purification kit according to the manufacturer’s protocol. The recombinant immunotoxin DNA region was sequenced using pET22b sequencing forward primer 5′-TAATACGACTCACTATAGGG-3′. Sequencing results were aligned using the Clustal Omega Multiple Alignment from BioEdit version 7.1.9 (Tom Hall, Fort Worth, TX, USA). The method for protein expression, isolation, affinity purification and refolding of recombinant immunotoxin was carried out according to scFv-eGFP protein as mentioned above.

**Table 2:** PCR primers for the amplification of recombinant immunotoxin.

Primer	Oligonucleotide sequence (5′–3″)
SCFV.FOR1 SCFV.REV1 MHALT1.FOR1 MHALT1.REV1	*Primers for scFv-HALT-1* GAATAAG**CCATGG**CT*CATCATCATCATCATCAT*ATGGCCCAGGTCCAACTGCAGCAGCC agaaccaccacctccagaaccaccaccaccagatccaccaccaccagaTTTTATTTCCAACTTTGTCC tctggtggtggtggatctggtggtggtggttctggaggtggtggttctGCAGCTTTAGGAGTTATAGC GTTGCCCTCGAGTCATCCAGAAAAAATAACTTTGA
MHALT1.FOR2 MHALT1.REV2 SCFV.FOR2 SCFV.REV2	*Primers for HALT-1-scFv* GAATAAG**CCATGG**CTCATCATCATCATCATCATGCAGCTTTAGGAGTTATAGC agaaccaccacctccagaaccaccaccaccagatccaccaccaccagaTCCAGAAAAAATAACTTTGA tctggtggtggtggatctggtggtggtggttctggaggtggtggttctATGGCCCAGGTCCAACTGCAGCAGCC GTTGCCCTCGAGTCATTTTATTTCCAACTTTGTCCC

**Note:**

Bold letters, *Nco*I restriction site; Underlined letters, *Xho*I restriction site; Italised letters, 6x His tag; Lower case letters, peptide linker sequence to generate a peptide linker fragment.

### SDS-PAGE and Western blot

Protein samples were loaded into 12% (w/v) sodium dodecyl sulfate (SDS) polyacrylamide gel and ran at 100 V for 100 min. The gel was stained with Coomassie blue (0.1% Coomassie blue, 45% methanol and 10% glacial acetic acid) and de-stained with 40% methanol and 10% glacial acetic acid solution overnight. The protein was transferred to a polyvinylidene fluoride (PVDF) membrane (Thermo Fisher, Waltham, MA, USA) for western blot analysis using a wet transfer system (Bio-rad, Irvine, CA, USA) for 90 min at 80 V in transfer buffer. The membrane was blocked with 1% (w/v) BSA in PBS for 1 h at RT to block protein-free binding spaces on the PVDF. Membrane was then incubated with HRP-anti-E tag antibody (Abcam, Cambridge, England, UK) (1:1,000) or His-detector nickel-HRP conjugate (KPL, Gaithersburg, MD, USA) (1:10,000) in 1% (w/v) BSA for 1 h with agitation at RT. The membrane was washed three times with 1X TBST buffer for 5 min and developed with TMB substrate (KPL, Gaithersburg, MD, USA). Bands were viewed with GS-800 Calibrated Imaging Densitometer (Bio-rad, Irvine, CA, USA).

### MTT cell viability assay

MTT assay was performed as described by Ng et al. (2019) with some modifications to determine the cytotoxic effects of recombinant immunotoxins using HCT-116 (*ATCC*^®^ CCL-247^™^), SW-480 (*ATCC*^®^ CCL-228^™^), and normal human dermal fibroblast (*ATCC*^®^ PCS-201-012^™^). Cell lines were prepared into cell suspensions with a density of 10,000 cells/well and incubated for 24 h to allow cell recovery and adherent to the wells. Different concentrations of recombinant immunotoxins (0, 5, 10, 15, 20, 25 and 30 μg/mL), mHALT-1 (0, 5, 10, 15, 20 and 25 μg/mL), anti-mKRAS G12V-34 scFv (0, 5, 10, 15, 20, 25 and 30 μg/mL) and glycerol (1%, 2.5%, 5.0%, 7.0%, 10% and 15%) diluted in DMEM media were added into respective wells and incubated at 37 °C for 48 h. The wells with media only without cells served as a negative control, while wells treated with 5% (v/v) dimethyl sulfoxide (DMSO) served as a positive control. After removing the media, 50 μL of 5 mg/mL MTT solution was added into the wells and incubated at 37 °C for another 3 h. The resultant purple formazan crystals were dissolved in 200 μL of DMSO. The absorbance intensity was measured by FLUOstar^®^ Omega (BMG Labtech, Germany) at 570 nm. All assays were performed in triplicates, and the relative cell viability was expressed as a percentage relative to untreated controls. The IC_50_ values were calculated in μg/mL or %, using Microsoft Excel. Differences between HCT116 or SW-480 treated cells against NHDF treated cells were analyzed using paired Student’s *t*-test. Significance was set at *p* < 0.05 (*) and *p* < 0.01 (**) for all tests.

### Cell-based ELISA assay

Cell-based ELISA assay was used to determine the binding activity of the refolded recombinant immunotoxins against colorectal cancer cells that expressed G12V, G13D or wt KRAS antigens. SW-480, HCT 116 and NHDF cell lines were seeded in 96-wells plates at 20,000 cells/well and incubated for 24 h. Cells were harvested after treatment with 0, 10 and 20 μg/mL of recombinant immunotoxins for 3 h. To identify background signals, non-coated wells with immunotoxin and blank with cell-coated wells without immunotoxin (untreated cells) were used as negative controls. Following three times of PBS washings, the cells were fixed with 4% (v/v) paraformaldehyde and incubated for 15 min at RT. After washing three times with PBS, the cells were blocked with 2% (w/v) BSA for 1 h to reduce non-specific binding. His-detector nickel-HRP conjugate (KPL, Gaithersburg, MD, USA) diluted at 1/10,000 in 1% (w/v) BSA (100 μL) was added, and the plates were incubated for 1 h at RT. The plates were washed three times with 0.05% (v/v) PBST. A hundred microliters of TMB substrate was added and the reaction was stopped by adding 50 μL of 1M H_2_SO_4_ per well. The OD_450_ was determined using FLUOstar^®^ Omega (BMG Labtech, Germany). All samples and controls were expressed as net absorbance by subtracting the mean absorbance for non-coated wells with immunotoxins and untreated cells. The relative absorbance at 450 nm was presented as mean values ± standard deviation. Paired student’s *t*-test was used to analyze differences between concentrations (0 μg/mL and 10 μg/mL or 20 μg/mL) and cell types (NHDF and HCT 116 or SW-480 cells). Significance was set at *p* < 0.05 (*) for all tests.

### Immunofluorescences staining assay

Immunofluorescence staining assay was carried out to identify the cellular localization upon binding of scFv using HCT-116, SW-480 and normal human dermal fibroblast cells. Cell lines were prepared into 300 µL of cell suspensions with a density of 50,000 cells/mL and incubated in three separate µ-Slide 8-well chambers (Ibidi, Germany) until confluency of 90% was achieved. The wells were washed three times with PBS to remove the media and different IC_50_ of purified scFv-eGFP proteins. After 24 h of incubation at 37 °C, cells were washed three times with PBS and fixed with 150 μL of 2% paraformaldehyde in PBS. Permeabilization was performed by incubating the cells with 150 μL of 0.1% (v/v) Triton X-100 for 20 min. The wells were washed three times with PBS, and 100 μL of 300 nM DAPI (4′,6-diamidino-2-phenylindole) stain solution was added. After incubation for 20 min in the dark, the cells were washed three times with PBS. All images were captured and analyzed using a fluorescence microscope Axio Vert A1 (Carl Zeiss, Germany), under 630× magnification and enhanced using ZEISS ZEN software. Both 488 and 405 nm laser lines were used for the excitation of eGFP and DAPI, respectively.

## Results

### Construction of the anti-mKRAS scFv library

**Figure 1:**
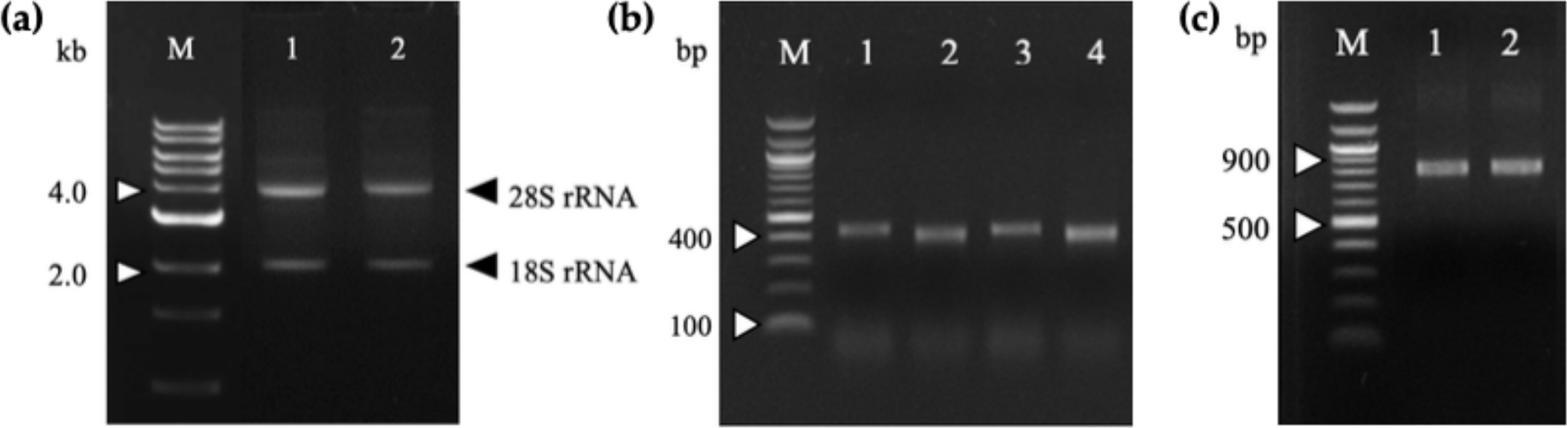
Agarose gel electrophoresis of total RNA and PCR-amplified products. (A) Total RNA extracted from 164-D (lane 1) and 68-V (lane 2) immunized mice splenocytes were electrophoresed on a 1.2% (w/v) agarose gel. The 18S and 28S rRNA bands are indicated (arrowheads). Lane M shows the Quick Load 1 kb DNA ladder (NEB, Hitchin, UK). (B) PCR amplification of the V_H_ and V_L_ fragments for 164-D and 68-V which are sized about 400 bp (Lane 1, 3) and 370 bp (lane 2, 4), respectively. Lane M shows the NEB Quick-Load 100 bp DNA ladder (NEB, Hitchin, UK). (C) SOE-PCR amplification of scFv fragments of 164-D (lane 1) and 68-V (lane 2) are about 750 bp. Lane M shows the NEB Quick-Load 100 bp DNA ladder (NEB, Hitchin, UK).

Total RNA was extracted from mice splenocytes immunized with G13D and G12V mutated *KRAS* mimotopes (termed 164-D and 68-V respectively) developed in our previous study [9] that showed high Ig titer immune responses. As shown in the gel electrophoresis of Fig. 1A, both total RNA samples were of good integrity, with two clear bands representing the 28S and 18S rRNA with an approximate ratio of 2:1. Total RNA samples were used immediately to generate cDNA for PCR amplification of immunoglobulin variable region (V_H_ and V_L_) genes. The anti-m*KRAS* 164-D and 68-V PCR products of V_H_ genes were about 400 bp (lane 1, lane 3) while V_L_ genes were about 370 bp (lane 2, lane 4), as shown in Fig. 1B. Both amplified V_H_ and V_L_ gene fragments were subjected to gel extraction and purified products were fused together by splicing by overlap extension (SOE)-PCR to produce a repertoire of scFv sequences. Figure 1C shows the assembled V_H_-linker-V_L_ scFv product of about 750 bp. The results indicated that both 164-D (lane 1) and 68-V (lane 2) scFv fragments were successfully amplified and incorporated with *Sfi*I and *Not*I restriction sites. A total of fifteen separate SOE-PCR rounds of scFv fragment pairings were performed, allowing random association of V_H_ and V_L_ to maximize the library size and diversity (data not shown). The assembled scFvs were purified from agarose gel and cloned into the *SfiI* and *NotI* restriction sites of pCANTAB5E phagemid vector and consequently transformed into the *E. coli* strain TG1 and confirmed by DNA sequencing. The average transformation efficiencies for pCANTAB5E:164-D and pCANTAB5E:68-V were 2.9 × 10^6^ and 3.4 × 10^6^ CFU/µg, respectively.

### Panning of scFv phage display library

The PEG-precipitated anti-m*KRAS* 164-D and 68-V scFv phages were subjected to three rounds of bio-panning against immobilized G13D and G12V control antigens (KRAS N-terminal protein, position 4–18), respectively. This step was carried out against control antigens instead of mimotopes, because the anti-m*KRAS* scFv antibodies would be used to recognize G12V/G13D k-ras antigens presented on the surface of cancer cells. For the 164-D library, the input titer was about 10^12^ CFU/mL, while the eluted phages titer increased at the second (10^5^) and third (10^6^) round of panning. For the 68-V library, the input titer was about 10^13^ CFU/mL, while the eluted phages titer increased at the second (10^6^) and third (10^7^) round of panning. These results indicated that more specific phage clones were binding to the respective peptide antigens during the third round of panning. As shown in Table 3, the phage recovery rate of second round panning showed about 10-fold increase in enrichment (10^−8^ to 10^−7^), which was increased approximately 60-fold by the third round (10^−8^ to 10^−6^). The overall enrichment was approximately 600-folds, which indicated successful enrichment of both 164-D and 68-V phages specifically binding to G13D and G12V antigens, respectively. After three rounds of binding selection, positive clones were further identified through phage-ELISAs.

**Table 3:** Panning efficiency of the 68-V and 164-D phage display library against G12V and G13D antigens, respectively.

Round of biopanning	Input (cfu/mL)		Output (cfu/mL)		Recovery rate (Output/Input ratio)	
	G13D	G12V	G13D	G12V	G13D	G12V
1^st^	3.6 × 10^12^	6.0 × 10^13^	2.1 × 10^5^	2.1 × 10^6^	5.8 × 10^−8^	3.5 × 10^−8^
2^nd^	1.7 × 10^12^	1.1 × 10^13^	7.5 × 10^5^	3.6 × 10^6^	4.4 × 10^−7^ 3.6 × 10^−6^	3.3 × 10^−7^
3^rd^	1.2 × 10^12^	2.8 × 10^13^	4.3 × 10^6^	6.5 × 10^7^		2.3 × 10^−6^

### Detection of phage-displayed anti-mKRAS recombinant scFvs

**Figure 2:**
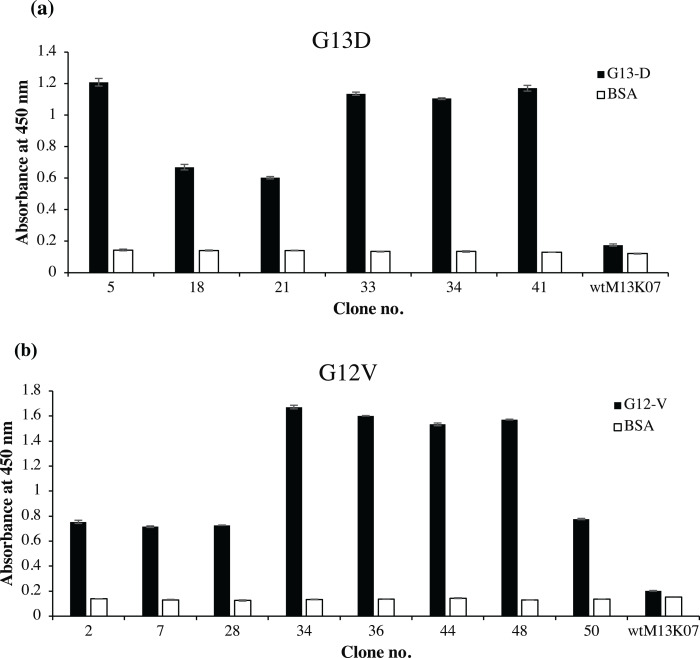
Identification of G12V and G13D m*KRAS* specific clones by phage-ELISA at 450 nm absorbance. After three rounds of panning, 50 individual randomly selected clones were infected with M13K07 helper phage to produce phage displayed scFv antibodies. Phage ELISA data for fourteen positive clones are shown. Clones that exhibited at least two times stronger ELISA signals than the BSA negative control (white bars) were deemed as specific clones. A total of (A) six positive clones of G13D and (B) eight positive clones of G12V were detected. Detection was performed using HRP-conjugated anti-M13 antibody and wt M13K07 helper phages were used as negative phage controls. Data were presented as mean values ± standard deviation indicated by error bars from triplicate experiments.

Fifty randomly picked single bacterial clones for each G13D and G12V were tested against its respective peptides. Figure 2 shows a representative of fourteen positive clones, six of which were positive to G13D and eight were positive to G12V peptide. Other bacterial clones gave absorbance values similar to those of the negative control wells coated with BSA (data not shown). Four positive clones of anti-m*KRAS* G13D scFv phage (G13D-5, G13D-33, G13D-34 and G13D-41) (Fig. 2A) showed the highest absorbance reading between 1.1 to 1.2, while a lower absorbance reading of 0.6 was obtained for G13D-18 and G13D-21. Background binding of the wt M13K07 negative control was minimal at an absorbance reading of 0.06. Meanwhile, four positive clones of anti-m*KRAS* G12V (G12V-34, G12V-36, G12V-44 and G12V-48) (Fig. 2B) showed the highest absorbance reading between 1.5 to 1.7. A lower absorbance reading of 0.7 was obtained for G12V-2, G12V-7, G12V-28 and G12V-50, but was nevertheless still two times higher than the control. Background binding of the wt M13K07 negative control was once again minimal with an absorbance reading of 0.05. Overall, both G12V and G13D scFv phages bound higher to their respective mutant *KRAS* antigens compared to BSA, suggesting that the isolated phage clones were selective towards the *KRAS* antigen. Collectively, the results generated from selection and phage ELISA indicated that high performance phage display libraries were successfully constructed which resulted in fourteen positive clones which were independently propagated and further confirmed by monoclonal ELISA.

### Sequence analysis of anti-mKRAS scFv clones

Sequencing of the 14 positive clones revealed that they corresponded to four distinct clones, with two anti-m*KRAS* G13D antibodies and another two anti-m*KRAS* G12V antibodies. For G13D clones, G13D-18 and G13D-21 shared identical sequences, while G13D-5, G13D-33, G13D-34, G13D-41 shared the same sequence. For G12V clones, G12V-2, G12V-7, G12V-28, G12V-50 had the same sequence, while G12V-34, G12V-36, G12V-44, G12V-48 shared the same sequence. This reflected the high amount of scFv phages enriched after three rounds of panning, suggesting that the lower affinity scFvs were eliminated after three rounds of panning. Thus, the scFv candidate list was further short-listed to four clones, two from each cluster for G13D and G12V, respectively: G12V-34, G12V-50, G13D-5 and G13D-18. NCBI IgBLAST (http://www.ncbi.nlm.nih.gov/igblast/) (Lefranc, 2001) was used to determine the number of amino acids, framework (FR), and complementary determining regions (CDRs) of the V_H_ and V_L_ domains. All the deduced amino acid sequences and CDRs are shown in Table 4. Taken together, the sequence homology analysis indicated that four V_H_ germline genes (V_H_1-4, V_H_1-22, V_H_1-56 and V_H_1-69) and four V_K_ germline genes (V_K_ 4-59, V_K_4-68, V_K_6-17 and V_K_12-46) were found in these murine immune antibody libraries. There were no sequence data that completely matched the variable regions of these antibodies in the database, suggesting the occurrence of new somatic hypermutations within the hypervariable CDRs.

**Table 4:** Amino acid sequences of G12V-34, G12V-50, G13D-5 and G13D-18 scFvs isolated from phage antibody libraries. CDR, complementarity determining regions; FR, framework regions.

scFv	FR1	CDR1	FR2	CDR2	FR3	CDR3	FR4
V_H_ sequences							
G12V-34	QVQLQQPGAELVRPGASVKLSCKTS	GYSFTSYW	MNWVKQRPGQGLEWIGY	INPSTGYT	EYNQKFKDKATLTADKSSSTAYMQLSSLTSEDSAVYYC	ARRDYRYFDV	WGAGTTVTVSS
G12V-50	QVQLKQSGAELVKPGASVKLSCKAS	GYTFTSYW	MHWVKQRPGQGLEWIGE	IDPSDSYT	NYNQKFKDKATLTVDKSSSTAYIQLSSLTSEDSAVYYC	ARGGYFDY	WGQGTTVTVSS
G13D-5	QVQLKQSGAELVKPGASVKLSCKAS	GYTFTSYW	MHWVKQRPGQGLEWIGE	IPSDSYT	NYNQKFKDKATLTVDKSSSTAYIQLSSLTSEDSAVYYC	ARGGYFDY	WGQGTTVTVSS
G13D-18	GSELVRPGASVKLSCKAS	GYTFTNYW	IHWVKQRPGQGLEWIGN	VYPGTGTT	YYDEKFKSKASLTVDTSSSTAFMQLSSLTSEDSAVYYC	TRIDYESLYFGV	WGAGTTLTVSS
V_L_ sequences							
G12V-34	DIVMTQSPALMSAALGERVTMTCSAS	SSVSY	MHWYQQKSGTSPKRWIY	DTS	KLASGVPARFSGSGSGTSYSLTISSMEAEDAATYYC	QQWSSNPFT	FGSGTKLEIK
G12V-50	DIVLTQSHKFICSSVGDRVSITCKAS	QDVSTA	VAWYQRKPGQSPKLLIY	SAS	YRCTGVPDRFTGSGSGTDYTLTISSVQAEDLALYYC	QQHYSTPFT	FGSGTKLEIK
G13D-5	DIVLTQSPALMSASPGEKVTMTCSAS	SSVSY	MYWYQQKPRSSPKPWIY	LTS	NLASGVPARFSGSGSGTSYSLTISSMEAEDAATYYC	QQWSNNPLT	FGAGTKLELK
G13D-18	DIQLTQSPASLSASVGETVTITCRAS	ENIYSY	LAWYQQKQGKSPQLLVY	AAT	NLADGVPSRFSGSGSGTQYSLKINSLQSEDFGSYYC	QHFWGTPFT	FGSGTKLEIK

### Expression and detection of soluble anti-mKRAS scFv antibodies by monoclonal-ELISA

**Figure 3:**
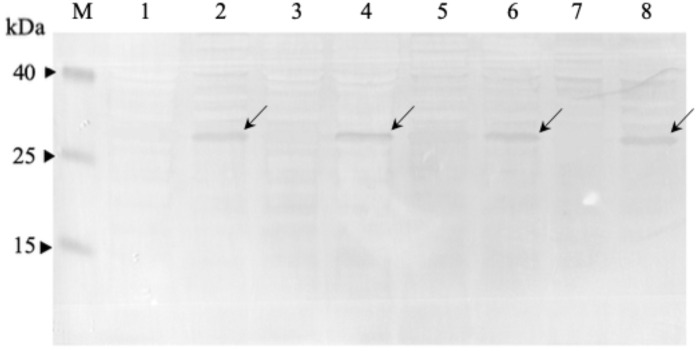
Western immunoblot analysis of anti-m*KRAS* scFvs in periplasmic fractions. Fractions were resolved on 12% (w/v) SDS-PAGE, immunoblotted and reacted with anti-E-tag antibody (1:1,000). Lane M, Thermo Fisher Spectra^™^ Multicolor low range protein ladder; Lane 1, IPTG-uninduced anti-m*KRAS* G13D-5 scFv; Lane 2, IPTG- induced anti-m*KRAS* G13D-5 scFv; Lane 3, IPTG-uninduced anti-m*KRAS* G13D-18 scFv; Lane 4, IPTG-induced anti-m*KRAS* G13D-18 scFv; Lane 5, IPTG-uninduced anti- m*KRAS* G12V-34 scFv; Lane 6, IPTG-induced anti-m*KRAS* G12V-34 scFv; Lane 7, IPTG-uninduced anti-m*KRAS* G12V-50 scFv; Lane 8, IPTG-induced anti-m*KRAS* G12V-50 scFv. Arrows indicate the presence of a 30 kDa band of anti-m*KRAS* scFv.

**Figure 4:**
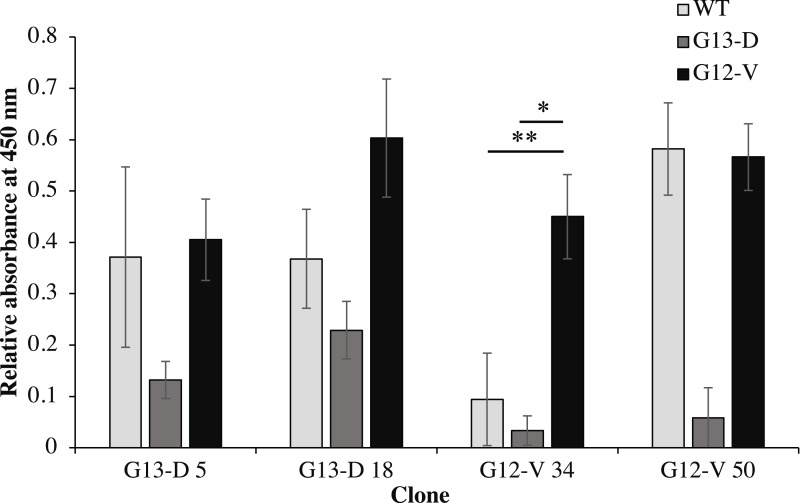
Cross-reactivity of soluble anti-m*KRAS* scFvs with control K-ras (G13D, G12V) and wildtype K-ras antigens in monoclonal-ELISA. Four clones (G13D-5, G13D-18, G12V-34 and G12V-50) were tested for binding specificity towards wt (light grey bars), G13D (dark grey bars) and G12V (black bars) control peptides. Wells were coated with 10 μg/mL of each antigen and bound antibodies were detected with anti E-tag antibodies (1:5,000). All values were presented as mean values ± standard deviation against background signals from plates without the addition of sera and non-coated wells, measuring at 450 nm wavelength. Significant differences were denoted as *p*-values < 0.05 (*) and <0.01 (**) when compared against G12V or G13D antigen controls.

Four clones (G12V-34, G12V-50, G13D-5, G13D-18) which were positive in phage-ELISA were selected for scFv expression in *E. coli* HB2151 cells. Expression of anti-m*KRAS* scFv proteins by the lac promoter was induced in the presence of 1 mM IPTG for 4 h at 30 °C. An anti-m*KRAS* scFv band at 30 kDa was detected using anti-E tag HRP-conjugated antibodies, further confirmed its expression in the periplasmic fraction (Fig. 3). No bands were observed in the uninduced periplasmic fraction. The specificity of soluble anti-m*KRAS* scFvs expressed in the periplasmic space was tested with a monoclonal-ELISA assay using anti-E-tag antibodies. Antigen controls (G13D, G12V) and wildtype K-ras antigens were employed to investigate the specificity of anti-m*KRAS* scFvs. ELISA results showed that both G13D clones (G13D-5, G13D-18) and G12V clones (G12V-34 and G12V-50) bound specifically to G13D and G12V antigens, respectively. The OD_450_ readings of each scFv antibody are as shown in Fig. 4, and the absorbance reading indicated a higher level for G12V- 50 at 0.566, followed by G12V-34 at 0.459, G13D-18 at 0.229, and G13D-5 at 0.132. Additionally, cross-reactivity of scFv antibodies towards wildtype and G13D/G12V mutated *KRAS* antigens were also evaluated. Although G13D clones showed binding affinity towards the G13D antigen, it also showed cross-reactivity towards wildtype (1-fold) and G12V (2- fold), which was undesirable. Similarly, while G12V-50 clone exhibited high specificity towards G12V, it was also found to highly cross-react with wildtype *KRAS* antigens at the same absorbance reading of 0.582 with G12V. Only the G12V-34 clone showed specific reactivity to G12V peptide, with weak cross-reactivity to wildtype (*p* = 0.005) and G13D (*p* = 0.01).

### Construction of recombinant immunotoxins

**Figure 5:**
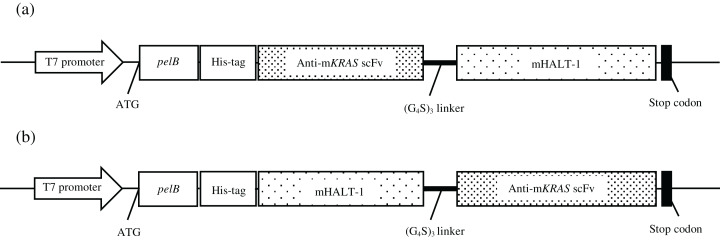
Constructs of recombinant immunotoxin anti-m*KRAS* scFv with mHALT-1. Schematic structure of the recombinant immunotoxins in pET22b expression vector. m*KRAS* scFv and HALT-1 were connected via the glycine serine peptide linker. (A) scFv-mHALT-1: N-terminal scFv and C-terminal mHALT-1. (B) mHALT-1-scFv: N-terminal mHALT-1 and C-terminal scFv.

**Figure 6:**
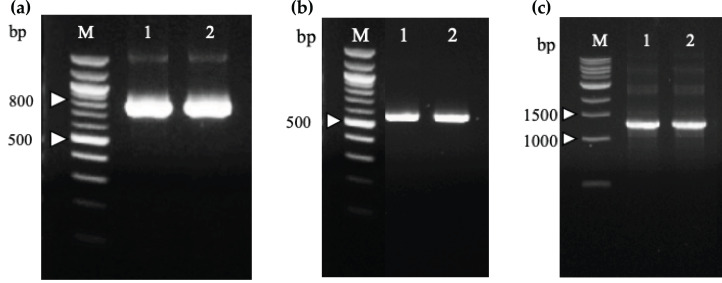
Agarose gel electrophoresis of PCR amplification of recombinant immunotoxins. (A) N-terminal (Lane 1) and C-terminal (Lane 2) anti-m*KRAS* G12V-34 scFv genes were amplified from plasmids pCANTAB5E:68-V 34, and are sized about 800 and 780 bp, respectively. Lane M shows the Quick-Load 100 bp DNA ladder (NEB, Hitchin, UK). (B) N-terminal (Lane 1) and C-terminal (Lane 2) mHALT-1 genes were amplified from pET28a:Y110A vector are sized about 570 and 550 bp, respectively. Lane M shows the Quick-Load 100 bp DNA ladder (NEB, Hitchin, UK). (C) SOE-PCR assembly of anti-m*KRAS* G12V-34 scFv and mHALT-1 into recombinant immunotoxins, scFv-mHALT-1 (Lane 1) and mHALT-1 scFv (Lane 2) resulting in 1,360 bp bands. Lane M shows the Quick-Load 1 kb DNA ladder (NEB, Hitchin, UK).

Two recombinant immunotoxin candidates were constructed with the scFv moiety arranged in two opposing orientations (Fig. 5) in order to determine which arrangement would be more effective. Figure 6A shows the amplified anti-m*KRAS* G12V-34 scFv PCR product with a band size of 801 bp (lane 1) for N-terminal scFv and a band size of 783 bp (lane 2) for C-terminal scFv. The mHALT-1 PCR product was successfully amplified (Fig. 6B) with the expected 576 bp fragment (lane 1) for N-terminal mHALT-1, and 558 bp fragment (lane 2) for C-terminal mHALT-1. The fused SOE-PCR products of 1,359 bp are shown in Fig. 6C. Both scFv-mHALT-1 (lane 1) and mHALT-1-scFv (lane 2) were successfully amplified with *Nco*I and *Xho*I restriction sites incorporated for subsequent cloning into pET22b vector. Positive transformants in *E. coli* BL21(DE3) cells were screened by cPCR and confirmed by DNA sequencing (data not shown).

### Expression, purification and refolding of recombinant immunotoxins

**Figure 7:**
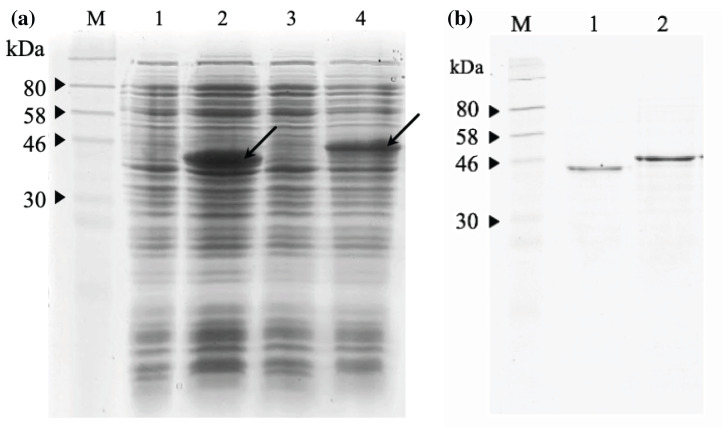
SDS-PAGE analysis of recombinant immunotoxins. (A) Protein expression was induced with 1 mM IPTG for 3 h at 30 °C. The samples were collected before and after induction. The cell pellets were resuspended in 2X Laemmli sample buffer and heated to denature. After centrifugation at 10,000*g* for 2 min, the supernatant was loaded on 12% (w/v) SDS- PAGE and stained with Coomassie brilliant blue. The IPTG-induced scFv-mHALT-1 (Lane 2) and mHALT-1 scFv band (Lane 4) at 44.4 kDa and 46.8 kDa, respectively, are shown with arrows. Uninduced scFv-mHALT-1 and mHALT-1-scFv were loaded on Lane 1 and Lane 3, respectively. (B) ****Denatured recombinant immunotoxins were refolded by step-wise dialysis using Snakeskin dialysis tubing (MWCO: 10 K) with decreasing concentrations of urea (6 M, 4 M, 2 M, 1 M) and final dialysis in PBS containing 25% glycerol. The refolded recombinant immunotoxins scFv-mHALT-1 (Lane 1) and mHALT-1-scFv (Lane 2) were loaded on 12% SDS-PAGE, and proteins were stained with Coomassie Brilliant Blue. Lane M shows the pre-stained broad range protein marker (NEB, Hitchin, UK).

The 12% SDS-PAGE electrophoresis results (Fig. 7A) indicated that both scFv-mHALT-1 and mHALT-1-scFv was successfully expressed in *E. coli* BL21(DE3) with an intense band at ~44.4 kDa and a moderate intense band at ~46.8 kDa, respectively. Variations in size were attributed to the failure of *pel*B leader sequence (2.4 kDa) to be cleaved off. Since both were expressed as insoluble recombinant proteins, inclusion bodies were isolated by ultrasonication, and the pellet fraction was washed with urea and Triton X-100 to remove proteinaceous contaminants that could affect protein refolding yields, followed by solubilization in chaotropic reagents such as urea. The solubilized inclusion bodies of scFv-mHALT-1 and mHALT-1-scFv were further purified by immobilized affinity chromatography using Ni-NTA resin and eluted by lowering the pH from 7.8 to 4.5. The purified and refolded recombinant immunotoxins scFv-mHALT-1 (Lane 1) and mHALT-1-scFv (Lane 2) of ~44.4 kDa and ~46.8 kDa respectively, is as shown in Fig. 7B, respectively. The final yield of scFv-mHALT-1 and mHALT-1-scFv immunotoxins were 49 μg/mL and 108 μg/mL with the recovery of 7.0% and 24.0%, respectively.

### Recombinant immunotoxins exhibit cytotoxic effects against KRAS-positive cancer cells

**Figure 8:**
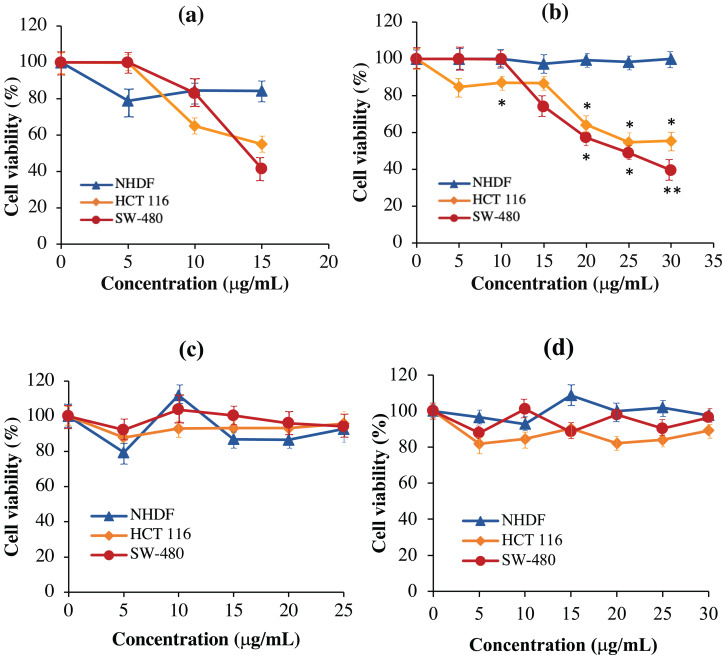
Normalized cell viability (%) of NHDF, HCT 116 and SW-480 cells treated with recombinant immunotoxins following 48-h of exposure as determined by MTT cytotoxicity assays. NHDF (blue line), HCT 116 (orange line), and SW-480 (red line) cells were plated at a density of 1 × 10^4^ cells/well and treated with (A) scFv-mHALT-1, (B) mHALT-1-scFv, (C) mHALT-1 and (D) ****anti-m*KRAS* G12V-34 scFv. Each point represents the mean ± standard deviation of three replicates, where significant differences were denoted as *p*-value < 0.05 (*) and <0.01 (**) when compared against NHDF treated cells.

As shown in Fig. 8A, the scFv-mHALT-1 immunotoxin treated HCT 116 and SW-480 cells were killed at an IC_50_ of 16.33 μg/mL and 15.60 μg/mL, respectively. As the concentration of scFv-mHALT-1 immunotoxin reached 15 μg/mL, the glycerol solvent percentage was also concurrently increased to 7.6%. Based on glycerol solvent controls, concentrations above 7.6% were shown to affect cell viability, and the absorbance readings recorded from this point onwards were no longer reliable. It is also notable that minimal cytotoxic effects of scFv-mHALT-1 immunotoxin were observed on NHDF cells, which concurred with the fact that NHDF cells do not express the G12V k-ras antigen. In Fig. 8B, mHALT-1-scFv immunotoxin exhibited cytotoxic effects on SW-480 cells and also HCT 116 cells but with higher IC_50_ values of 25.39 μg/mL and 31.91 μg/mL respectively compared to the scFv-mHALT-1 immunotoxin. Compared to scFv-mHALT-1, mHALT-1-scFv contains lower glycerol concentrations, because higher concentration of mHALT-1-scFv was obtained following refolding steps. At a 30 μg/mL concentration of mHALT-1-scFv, approximately 6.9% of glycerol content was present. This was a concentration that did not affect cell viability. No cytotoxicity effects were observed for mHALT-1-scFv immunotoxin treated NHDF cells even at concentrations as high as 30 μg/mL, which was a desirable observation. When comparing against mHALT-1 and anti-m*KRAS* G12V-34 scFv standalone controls (Figs. 8C & 8D), the mHALT-1-scFv immunotoxin was shown to re-exhibit its cytotoxicity effects on *KRAS*-positive cancer cells with minimal killing of *KRAS*-negative cells, thus reinforcing the notion that the scFv moiety acted as a targeting tool for the mHALT-1 toxin.

### Determinaton of recombinant immunotoxin specificity using cell-based ELISA

**Figure 9:**
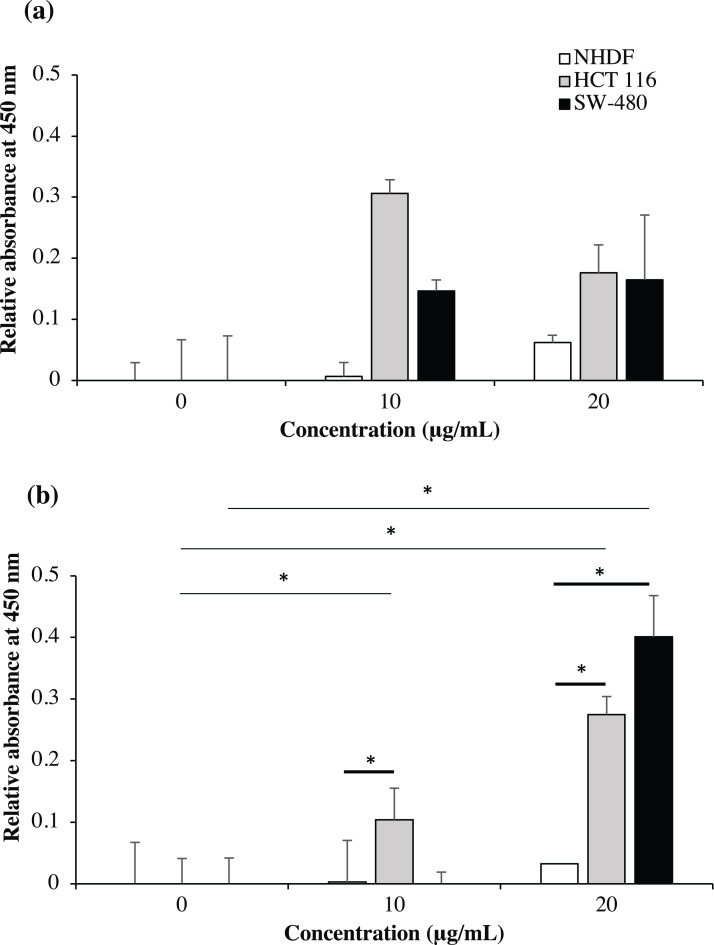
Cell-based ELISA with NHDF (wt *KRAS*-negative), HCT 116 (G13D *KRAS*-positive) and SW-480 (G12V *KRAS*-positive) cells treated with recombinant immunotoxins. NHDF (white bars), HCT 116 (grey bars) and SW-480 (black bars) cell lines were treated with 0, 10 and 20 μg/mL of scFv-mHALT-1 (A) and mHALT-1-scFv (B). Wells were formaldehyde-fixed with cells and bound immunotoxins were detected by using his-tag nickel-HRP conjugate (1:10,000). The relative absorbance at 450 nm was presented as mean values ± standard deviation against background signals from non-coated wells with immunotoxins and untreated cells. Error bars represent the standard error of the mean from experimental replicates. Significant differences were denoted as *p*-values < 0.05 (*) when compared against untreated and NHDF cell controls.

As shown in Fig. 9, no significant binding for scFv-mHALT-1 (*p* = 0.473) and mHALT-1-scFv (*p* = 0.437) immunotoxins were detected towards NHDF cells (wt*KRAS*-negative), even up to concentrations of 20 μg/mL. This observation was expected with *KRAS*-negative cell lines that do not express the G12V k-ras antigen. Binding of scFv-mHALT-1 immunotoxin against HCT 116 (G13D *KRAS*-positive) were not significantly different (*p* = 0.080), with absorbance reading at 0.306 (10 μg/mL) and 0.176 (20 μg/mL). Similar insignificant values (*p* = 0.203 and 0.114) were observed for SW-480 cells (G12V *KRAS*-positive) when compared to untreated controls. These results were consistent and supported data obtained in MTT cytotoxic assays. For mHALT-1-scFv immunotoxin, significant differences (*p* = 0.050) were observed in HCT 116 treated cells when compared to untreated cells. A significant (*p* = 0.032) difference was also observed when SW-480 cells was treated with 20 μg/mL of mHALT-1-scFv. Collectively, when comparing between *KRAS*-negative (NHDF cells) and *KRAS*-positive (HCT 116 or SW-480) cells, both *KRAS*-positive cell lines showed significantly higher differences at *p* = 0.028 and 0.034. These results demonstrated that the mHALT-1-scFv immunotoxin was reacting with both *KRAS* G12V (SW-480) and *KRAS* G13D (HCT 116) expressing cell lines, but not for the *KRAS*-negative cell line (NHDF). This suggested that the anti-m*KRAS* G12V scFv targeting moiety could differentiate between wt and mt *KRAS* cells, but not between *KRAS* subtype G12V and G13D.

### Immunofluorescence localization of scFv-eGFP

**Figure 10:**
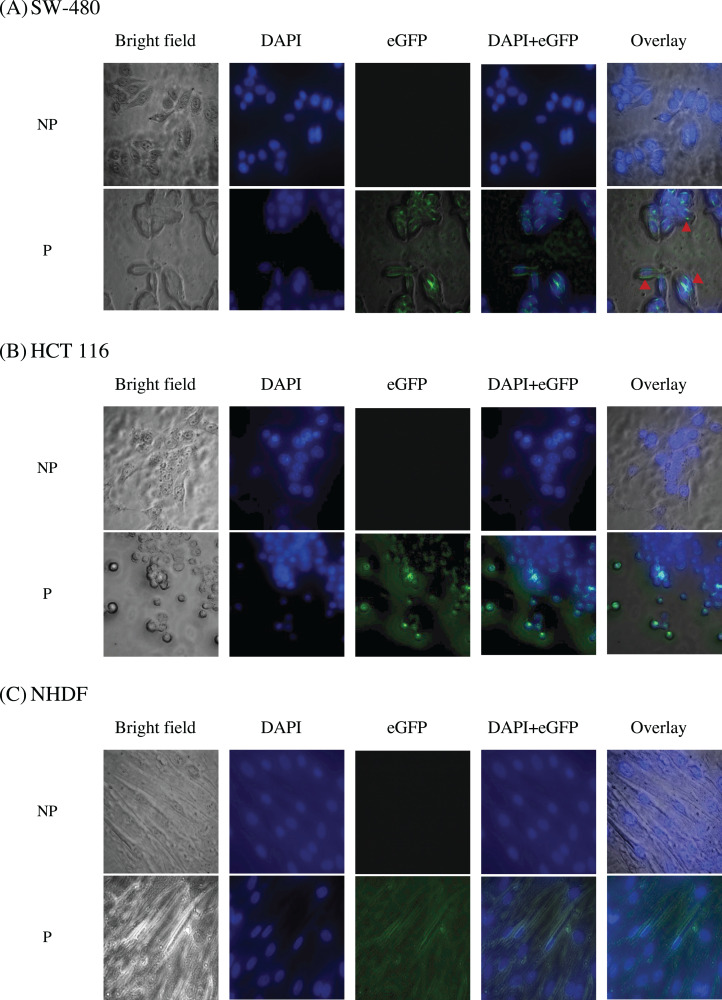
Immunofluorescence staining of (A) SW-480 (G12V *KRAS*-positive), (B) HCT 116 (G13D *KRAS*-positive) and (C) NHDF (wt *KRAS*-negative) cells. Blue fluorescence denotes nuclei stained with DAPI, while green fluorescence represents the binding localization of scFv via eGFP. Images were obtained on an Axio Vert A1 under 630× magnification and arranged with ZEISS Zen software. Red arrows indicate scFv binding. NP, non-permeabilization; P, permeabilization.

To study the localization of scFv via eGFP, immunofluorescence staining was performed on NHDF cells (wt*KRAS*-negative), SW-480 cells (G12V *KRAS*-positive), and HCT 116 (G13D *KRAS*-positive) cells as shown in Fig. 10. The cell lines were incubated with different IC_50_ of scFv-eGFP for 24 h followed by countered stained with DAPI. As shown in Fig. 10, blue fluorescent cells were observed due to dsDNA’s binding to AT regions in all the DAPI-stained nuclei. Green fluorescent was observed in all the permeabilized cells treated with the scFv-eGFP protein with different localization. Of note, green fluorescent was found predominantly on the inner membrane of SW-480 cells, while the scattering of green fluorescent can be observed in the cytoplasm of HCT 116 and NHDF cells. This is suggesting the binding of scFv was specifically tethered to the cell membranes of SW-480 that express G12V k-ras antigen.

## Discussion

Phage display antibody technology offers a convenient tool for isolation of high affinity and specific antibody fragments, which can be applied clinically as a potential targeting vehicle for immunotherapy, diagnosis, and drug delivery purposes in cancers. While, *KRAS* is known to be expressed and anchored to the inner membrane of cells, there were several reports highlighting the MHC-I presentation of mutated *KRAS* on the cell surface, thus rendering it a plausible and druggable target using immunotherapeutic approaches (June, 2016; Tran et al., 2016). Therefore, the present study was aimed at identifying anti-m*KRAS* antibodies for *KRAS* colorectal cancer by utilizing phage display libraries to screen for antibodies that selectively target G12V and G13D *KRAS*-positive colorectal cancer cells.

ScFv constructs were generated by randomly joined V_H_ and V_L_ through SOE-PCR. Such random pairings generated a high diversity of scFv fragments, yielding antibodies with novel specificities alongside natural combinations in vivo by the murine immune system (Griffiths & Duncan, 1998). The 15-amino acid linker was selected to assemble the V_H_ and V_L_ gene due to several reasons, including the small size of glycine and serine amino acids, which allows the mobility of the connecting functional domains, reduces unfavorable interactions between the linker and protein moieties by the formation of hydrogen bonds with water molecules, and to provide stability at levels comparable to native antibodies (Takkinen et al., 1991; Chen, Zaro & Shen, 2013).

The combinatorial library for mice V_H_ and V_L_ chains were effectively and specifically amplified from 164-D and 68-V immunized mice splenocytes, and as a result, a large pool of scFvs available for affinity selection by bio-panning was constructed with a complexity of 3.4 × 10^6^ and 2.9 × 10^6^ independent transformants, respectively. The panning protocol involved three rounds of selection against plates coated with G12V and G13D antigens, with an enrichment factor similar to those previously reported (Salvatore et al., 2002; Deng et al., 2005; Gong et al., 2016). Despite a moderate library size, this study demonstrated that utilization of this immunized library in conjunction with an optimized panning protocol was sufficient to identify specific *KRAS* G12V and G13D anti-m*KRAS* scFvs.

The successful isolation of specific scFv binders to m*KRAS* G12V antigen is analogous to a guidance system that is able to carry a very lethal toxin load, thus enabling the selective killing of *KRAS*-positive colorectal cancer cells while sparing normal cells. Consequently, the anti-m*KRAS* G12V antibody developed through this study was fused with a mutated pore forming *Hydra magnipapillata* toxin, giving rise to a total of two recombinant immunotoxins in two orientations (scFv-mHALT-1 and mHALT-1-scFv). Although the soluble expression of immunotoxins is preferable, the high yield of refolded immunotoxins from inclusion bodies is a promising approach to isolate high purity immunotoxins. Previous studies have shown that his-tag recombinant proteins can be purified efficiently under denaturing conditions (Lemercier, Bakalara & Santarelli, 2003; Arnau, Lauritzen & Pedersen, 2006). Alternatively, functional cytoplasmic expression can be attempted using mutant strains with a more oxidized cytoplasm or by fusing to cytoplasmic localization proteins. Also, the use of yeast or mammalian host cells can be an alternative strategy to express secreted and soluble recombinant immunotoxins, thus eliminating the need for in vitro refolding (Della Cristina et al., 2015).

MTT assay results of scFv-mHALT-1 and mHALT-1-scFv recombinants showed that these immunotoxins could elicit cytotoxic effects on *KRAS*-positive cancer cells without affecting normal fibroblast cells. Three to four HALT subunits are required to form pores that cause cell lysis, thus higher concentrations of immunotoxins are required to kill mutated *KRAS* cells in this study as compared to other immunotoxin studies showing lower IC_50_ values within the ng/mL range (Hashimi et al., 2013; Liew et al., 2015; Xie et al., 2017). Based on these results, cell-based ELISA assay further confirmed the binding specificity of mHALT-1-scFv immunotoxin towards G12V *KRAS* SW-480 and G13D *KRAS* HCT 116 cancer cells.

While data obtained from this proof of concept study is promising, several concerns on its application should also be highlighted. This includes vascular leak syndrome and other negative effects of immunotoxins on immune cells that present mutated KRAS antigens such as dendritic cells. Such APCs typically overexpress checkpoint molecules to deter CD8 killing, but would have no protective effects against immunotoxins (Li et al., 2017; Wei, Duffy & Allison, 2018). Meanwhile, the immunogenicity of the HALT can be reduced by deimmunizing the toxin moiety through point mutations on epitope regions (Schmohl et al., 2018).

Therefore, additional validation experiments including testing of immunotoxin stability, serum half-life and pre-clinical in vivo safety assessment are required. Continued efforts to produce soluble and humanized antibody versions from mouse scFv could further enhance its therapeutic potential.

## Conclusions

In conclusion, this work is the first to describe the development of recombinant immunotoxins to target *KRAS*-positive cancer cells coupled with a potent *Hydra* toxin. Hence, the development of mHALT-1-scFv immunotoxin is potentially useful as an immunotherapeutic application against *KRAS*-positive malignancies, thereby reducing dose-related toxicities and undesirable adverse side effects commonly seen in conventional non-targeted chemotherapeutic regimens. Additionally, the anti-m*KRAS* scFv can also be potentially developed as a predictive tool through histological, cytological or serological based applications for the detection of the G12V m*KRAS* genotypes during cancer screening or diagnosis. Various toxins, biomarkers or drugs can also be fused to this scFv sequence, thus unleashing vast opportunities for further downstream innovations.

## Supplemental Information

10.7717/peerj.11063/supp-1 Supplemental Information 1 Phage-ELISA Absorbance Readings.

10.7717/peerj.11063/supp-2 Supplemental Information 2 Monoclonal-ELISA Absorbance Readings.

10.7717/peerj.11063/supp-3 Supplemental Information 3 Cell Viability Assay Absorbance Readings.

10.7717/peerj.11063/supp-4 Supplemental Information 4 Cell-based ELISA Assay Absorbance Readings.

10.7717/peerj.11063/supp-5 Supplemental Information 5 S5 DNA sequence for review. Chromatogram of pCANTAB5E:G12V-34, pCANTAB5E:G12V-50, pCANTAB5E:G13D-5, pCANTAB5E:G13D-18 DNA sequencing results.

10.7717/peerj.11063/supp-6 Supplemental Information 6 Full length gel images. Figures 1(a), 1(b), 1(c), 3, 6(a), 6(b), 6(c), 7(a), 7(b).

## Additional Information and Declarations

### DNA Deposition

The following information was supplied regarding the deposition of DNA sequences: The scFv G12V-34 sequence described here is available at GenBank: MT799824.

### Data Availability

The following information was supplied regarding data availability: Raw measurements are available in the Supplemental Files.
