# Supplementary material for: Development of a single-chain fragment variable fused-mutant HALT-1 recombinant immunotoxin against G12V mutated KRAS colorectal cancer cells
Source: PeerJ. 2021 Apr 15;9:e11063. doi: 10.7717/peerj.11063 (PMC8053384; doi:10.7717/peerj.11063)
Supplement: Supplemental Information 1 [file peerj-09-11063-s001.docx]

**PHAGE-ELISA ABSORBANCE READINGS**

**PHAGE-ELISA ABSORBANCE READINGS OF G12V CLONES AT OD_450_**

| Clone no. | 2 | 7 | 28 | 34 | 36 | 44 | 48 | 50 | wt M13K07 |
| --- | --- | --- | --- | --- | --- | --- | --- | --- | --- |
| Reading 1  Reading 2  Reading 3  Average  Standard deviation  *BSA-coated*  Reading 1  Reading 2  Reading 3  Average  Standard deviation | 0.743  0.751  0.768  0.754  0.013  0.139  0.138  0.138  0.138  0.001 | 0.712  0.723  0.712  0.716  0.006  0.129  0.128  0.135  0.131  0.004 | 0.725  0.728  0.728  0.727  0.002  0.121  0.131  0.125  0.126  0.005 | 1.655  1.678  1.682  1.672  0.015  0.131  0.135  0.135  0.134  0.002 | 1.601  1.601  1.604  1.602  0.002  0.138  0.136  0.135  0.136  0.002 | 1.545  1.532  1.523  1.533  0.011  0.141  0.147  0.145  0.144  0.003 | 1.575  1.569  1.571  1.572  0.003  0.130  0.131  0.131  0.131  0.001 | 0.780  0.771  0.779  0.777  0.005  0.135  0.135  0.137  0.136  0.001 | 0.201  0.206  0.201  0.203  0.003  0.152  0.151  0.156  0.153  0.003 |

**PHAGE-ELISA ABSORBANCE READINGS OF G13D CLONES AT OD_450_**

| Clone no. | 5 | 18 | 21 | 33 | 34 | 41 | wt M13K07 |
| --- | --- | --- | --- | --- | --- | --- | --- |
| Reading 1  Reading 2  Reading 3  Average  Standard deviation  *BSA-coated*  Reading 1  Reading 2  Reading 3  Average  Standard deviation | 1.221  1.181  1.225  1.209  0.024  0.142  0.141  0.151  0.145  0.006 | 0.651  0.685  0.675  0.670  0.017  0.140  0.145  0.139  0.141  0.003 | 0.600  0.612  0.598  0.603  0.008  0.140  0.140  0.143  0.141  0.002 | 1.139  1.145  1.125  1.136  0.010  0.135  0.136  0.132  0.134  0.002 | 1.103  1.111  1.101  1.105  0.005  0.137  0.138  0.130  0.135  0.004 | 1.192  1.159  1.162  1.171  0.018  0.130  0.131  0.130  0.130  0.001 | 0.181  0.179  0.168  0.176  0.007  0.121  0.124  0.119  0.121  0.003 |
