# Supplementary material for: Development of a single-chain fragment variable fused-mutant HALT-1 recombinant immunotoxin against G12V mutated KRAS colorectal cancer cells
Source: PeerJ. 2021 Apr 15;9:e11063. doi: 10.7717/peerj.11063 (PMC8053384; doi:10.7717/peerj.11063)
Supplement: Supplemental Information 2 [file peerj-09-11063-s002.docx]

**MONOCLONAL-ELISA ABSORBANCE READINGS**

**MONOCLONAL-ELISA ABSORBANCE READINGS OF G12V-34 SCFV AT OD_450_**

| Peptide | WT | Non-coated | Blank | G13D | Non-coated | Blank | G12V | Non-coated | Blank |
| --- | --- | --- | --- | --- | --- | --- | --- | --- | --- |
| *Replicate 1*  Reading 1  Reading 2  Average  Standard deviation  Relative mean^a^  *Replicate 2*  Reading 1  Reading 2  Average  Standard deviation  Relative mean^a^  Average relative mean  Standard deviation | 0.306  0.261  0.284  0.032  0.078  0.450  0.419  0.435  0.022  0.111  0.094  0.090 | 0.07  0.062  0.066  0.006  0.070  0.089  0.080  0.013 | 0.144  0.136  0.140  0.006  0.262  0.226  0.244  0.025 | 0.177  0.180  0.179  0.002  0.030  0.229  0.227  0.228  0.001  0.038  0.034  0.029 | 0.071  0.061  0.066  0.007  0.083  0.081  0.082  0.001 | 0.085  0.081  0.083  0.003  0.112  0.105  0.109  0.005 | 0.684  0.676  0.680  0.006  0.437  0.817  0.827  0.822  0.007  0.463  0.450  0.082 | 0.067  0.075  0.071  0.006  0.087  0.079  0.083  0.006 | 0.158  0.187  0.173  0.021  0.295  0.258  0.277  0.026 |

^a^ Relative mean value is average value of each concentration subtracted with average of non-coated and blank

**MONOCLONAL-ELISA ABSORBANCE READINGS OF G12V-50 SCFV AT OD_450_**

| Peptide | WT | Non-coated | Blank | G13D | Non-coated | Blank | G12V | Non-coated | Blank |
| --- | --- | --- | --- | --- | --- | --- | --- | --- | --- |
| *Replicate 1*  Reading 1  Reading 2  Average  Standard deviation  Relative mean^a^  *Replicate 2*  Reading 1  Reading 2  Average  Standard deviation  Relative mean^a^  Average relative mean  Standard deviation | 0.836  0.765  0.801  0.050  0.546  0.976  0.899  0.938  0.054  0.618  0.582  0.090 | 0.120  0.111  0.116  0.006  0.076  0.087  0.082  0.008 | 0.144  0.135  0.140  0.006  0.245  0.231  0.238  0.010 | 0.270  0.285  0.278  0.011  0.058  0.378  0.380  0.379  0.001  0.058  0.058  0.059 | 0.091  0.097  0.094  0.004  0.080  0.079  0.080  0.001 | 0.123  0.129  0.126  0.004  0.247  0.236  0.242  0.008 | 0.798  0.855  0.827  0.040  0.559  0.946  0.911  0.928  0.025  0.573  0.566  0.065 | 0.089  0.071  0.080  0.013  0.079  0.089  0.084  0.007 | 0.177  0.198  0.188  0.015  0.278  0.265  0.272  0.009 |

^a^ Relative mean value is average value of each concentration subtracted with average of non-coated and blank

**MONOCLONAL-ELISA ABSORBANCE READINGS OF G13D-5 SCFV AT OD_450_**

| Peptide | WT | Non-coated | Blank | G13D | Non-coated | Blank | G12V | Non-coated | Blank |
| --- | --- | --- | --- | --- | --- | --- | --- | --- | --- |
| *Replicate 1*  Reading 1  Reading 2  Average  Standard deviation  Relative mean^a^  *Replicate 2*  Reading 1  Reading 2  Average  Standard deviation  Relative mean^a^  Average relative mean  Standard deviation | 0.737  0.569  0.653  0.119  0.237  0.815  0.992  0.904  0.125  0.506  0.371  0.176 | 0.116  0.108  0.112  0.006  0.077  0.089  0.083  0.008 | 0.311  0.298  0.305  0.009  0.289  0.340  0.315  0.036 | 0.448  0.439  0.444  0.006  0.144  0.498  0.411  0.455  0.062  0.120  0.132  0.036 | 0.115  0.105  0.110  0.007  0.080  0.076  0.078  0.003 | 0.200  0.179  0.190  0.015  0.246  0.267  0.257  0.015 | 0.897  0.813  0.855  0.059  0.440  0.754  0.715  0.735  0.028  0.371  0.405  0.079 | 0.105  0.100  0.103  0.004  0.090  0.070  0.080  0.014 | 0.315  0.311  0.313  0.003  0.287  0.281  0.284  0.004 |

^a^ Relative mean value is average value of each concentration subtracted with average of non-coated and blank

**MONOCLONAL-ELISA ABSORBANCE READINGS OF G13D-18 SCFV AT OD_450_**

| Peptide | WT | Non-coated | Blank | G13D | Non-coated | Blank | G12V | Non-coated | Blank |
| --- | --- | --- | --- | --- | --- | --- | --- | --- | --- |
| *Replicate 1*  Reading 1  Reading 2  Average  Standard deviation  Relative mean^a^  *Replicate 2*  Reading 1  Reading 2  Average  Standard deviation  Relative mean^a^  Average relative mean  Standard deviation | 0.978  0.765  0.872  0.151  0.285  0.876  0.956  0.916  0.057  0.451  0.368  0.096 | 0.111  0.101  0.106  0.007  0.121  0.111  0.116  0.007 | 0.451  0.510  0.481  0.042  0.351  0.347  0.349  0.003 | 0.671  0.545  0.608  0.089  0.219  0.655  0.623  0.639  0.023  0.239  0.229  0.056 | 0.187  0.199  0.193  0.023  0.156  0.123  0.140  0.023 | 0.180  0.212  0.196  0.023  0.245  0.276  0.261  0.022 | 0.987  1.010  0.999  0.016  0.491  1.240  1.110  1.175  0.092  0.716  0.603  0.115 | 0.178  0.189  0.184  0.008  0.134  0.147  0.141  0.009 | 0.298  0.351  0.325  0.037  0.324  0.314  0.319  0.007 |

^a^ Relative mean value is average value of each concentration subtracted with average of non-coated and blank
