# Supplementary material for: Development of a single-chain fragment variable fused-mutant HALT-1 recombinant immunotoxin against G12V mutated KRAS colorectal cancer cells
Source: PeerJ. 2021 Apr 15;9:e11063. doi: 10.7717/peerj.11063 (PMC8053384; doi:10.7717/peerj.11063)
Supplement: Supplemental Information 3 [file peerj-09-11063-s003.docx]

**MTT ASSAY ABSORBANCE READINGS OF NHDF TREATED CELLS**

**MTT ASSAY ABSORBANCE READINGS OF SCFV-MHALT-1 TREATED NHDF CELLS**

| Concentration (μg/mL) | | | | | | | | | |  |
| --- | --- | --- | --- | --- | --- | --- | --- | --- | --- | --- |
|  | Blank | 0 | 5 | 10 | 15 | 20 | 25 | 30 | DMSO | Camptothecin |
| *1^st^ Replicate*  Reading 1  Reading 2  Reading 3  Average  Minus blank^a^  Standard deviation  Cell viability  *2^nd^ Replicate*  Reading 1  Reading 2  Reading 3  Average  Minus blank^a^  Standard deviation  Cell viability  Average cell viability^c^  Standard deviation  Glycerol (%) | 0.043  0.042  0.041  0.042  0.056  0.051  0.057  0.055 | 0.281  0.361  0.258  0.300  0.342  0.054  100.0%  0.287  0.271  0.298  0.286  0.232  0.012  100.0%  100.0%  0.036  0.0 | 0.295  0.221  0.236  0.251  0.209  0.039  61.1%  0.225  0.226  0.222  0.224  0.170  0.002  73.3%  67.2%  0.029  2.5 | 0.261  0.216  0.244  0.240  0.198  0.023  57.9%  0.223  0.212  0.218  0.218  0.163  0.006  70.3%  64.1%  0.019  5.0 | 0.226  0.216  0.171  0.204  0.162  0.029  47.4%  0.209  0.229  0.211  0.216  0.162  0.011  55.2%  51.3%  0.021  7.6 | 0.172  0.169  0.187  0.176  0.134  0.010  39.2%  0.163  0.169  0.164  0.165  0.111  0.003  47.8%  43.5%  0.009  10.0^b^ | 0.141  0.140  0.141  0.141  0.099  0.001  28.9%  0.141  0.132  0.141  0.138  0.083  0.005  35.8%  32.4%  0.004  12.7^b^ | 0.136  0.142  0.131  0.136  0.094  0.006  27.4%  0.095  0.097  0.111  0.101  0.046  0.009  19.8%  23.6%  0.021  15.3^b^ | 0.109  0.117  0.114  0.113  0.071  0.004  20.8%  0.099  0.097  0.094  0.097  0.042  0.003  18.1%  19.5%  0.010  - | 0.321  0.300  0.302  0.308  0.266  0.012  77.7%  0.220  0.226  0.233  0.226  0.171  0.007  73.7%  75.7%  0.045  - |

^a^ Minus blank is average value subtracted with average of blank. Standard deviation is standard error calculate from the three readings.

Percentage of cell viability is calculated based on Equation 3.6.

^b^ Glycerol content that affect the cell viability.

^c^ Average cell viability (%) is average cell viability (%) of replicate 1 and replicate 2

**NORMALISED ABSORBANCE READINGS OF SCFV-MHALT-1 IMMUNOTOXIN TREATED NHDF CELLS**

| Concentration (μg/mL) | | | | | | | |
| --- | --- | --- | --- | --- | --- | --- | --- |
|  | 0 | 5 | 10 | 15 | 20 | 25 | 30 |
| Cell viability (%)  Dead cells (%)^a^  Glycerol (%)^b^  Dead cells (%)^c^  Normalised dead cells (%)^d^  Normalised cell viability (%)^e^ | 100.0  0.0  0.0  0.0  0.0  100.0 | 67.2  32.8  2.5  11.6  21.2  78.8 | 64.1  35.9  5.0  20.5  15.4  84.6 | 51.3  48.7  7.6  33.0  15.7  84.3 | 43.5  56.5  10.0  80.7  -24.3  100.0^f^ | 32.4  67.6  12.7  88.7  -21.0  100.0^f^ | 23.6  76.4  15.3  96.8  -20.4  100.0^f^ |

^a^ Dead cells (%) = 100% - % of cell viability

^b^ Percentage of glycerol in immunotoxins

^c^ Percentage of dead cells from glycerol treated cells from TABLE 7.1

^d^ Normalised dead cells (%) = a - c

^e^ Normalised cell viability (%) = 100% - d

^f^ Glycerol content that affect the cell viability

**MTT ASSAY ABSORBANCE READINGS OF MHALT-1-SCFV TREATED NHDF CELLS**

| Concentration (μg/mL) | | | | | | | | | |
| --- | --- | --- | --- | --- | --- | --- | --- | --- | --- |
|  | Blank | 0 | 5 | 10 | 15 | 20 | 25 | 30 | DMSO |
| *1^st^ Replicate*  Reading 1  Reading 2  Reading 3  Average  Minus blank^a^  Standard deviation  Cell viability  *2^nd^ Replicate*  Reading 1  Reading 2  Reading 3  Average  Minus blank^a^  Standard deviation  Cell viability  Average cell viability^c^  Standard deviation  Glycerol (%) | 0.043  0.042  0.041  0.042  0.056  0.051  0.057  0.055 | 0.297  0.253  0.314  0.288  0.246  0.031  100.0%  0.285  0.317  0.298  0.300  0.245  0.016  100.0%  100.0%  0.023  0.0 | 0.274  0.315  0.328  0.306  0.264  0.028  107.3%  0.284  0.294  0.318  0.299  0.244  0.017  99.6%  103.5%  0.021  1.2 | 0.262  0.233  0.287  0.260  0.218  0.027  88.6%  0.262  0.283  0.287  0.277  0.223  0.013  91.0%  89.8%  0.021  2.3 | 0.254  0.232  0.212  0.233  0.191  0.021  77.6%  0.254  0.272  0.262  0.263  0.208  0.009  84.9%  81.3%  0.022  3.5 | 0.221  0.262  0.221  0.235  0.193  0.024  78.5%  0.251  0.262  0.254  0.256  0.201  0.006  82.0%  80.3%  0.019  4.6 | 0.221  0.214  0.221  0.219  0.177  0.004  72.0%  0.251  0.254  0.251  0.252  0.197  0.002  80.4%  76.2%  0.018  5.8 | 0.200  0.277  0.219  0.232  0.190  0.040  77.2%  0.231  0.237  0.219  0.229  0.174  0.009  71.0%  74.1%  0.026  6.9 | 0.109  0.117  0.114  0.113  0.071  0.004  28.9%  0.099  0.097  0.094  0.097  0.042  0.003  17.1%  23.0%  0.010  - |

^a^ Minus blank is average value subtracted with average of blank. Standard deviation is standard error calculate from the three readings.

Percentage of cell viability is calculated based on Equation 3.6.

^b^ Glycerol content that affect the cell viability.

^c^ Average cell viability (%) is average cell viability (%) of replicate 1 and replicate 2

**NORMALISED ABSORBANCE READINGS OF MHALT-1-SCFV IMMUNOTOXIN TREATED NHDF CELLS**

| Concentration (μg/mL) | | | | | | | |
| --- | --- | --- | --- | --- | --- | --- | --- |
|  | 0 | 5 | 10 | 15 | 20 | 25 | 30 |
| Cell viability (%)  Dead cells (%)^a^  Glycerol (%)^b^  Dead cells (%)^c^  Normalised dead cells (%)^d^  Normalised cell viability (%)^e^ | 100.0  0.0  0.0  0.0  0.0  100.0 | 103.5  -3.5  1.2  7.3  -10.8  100.0 | 89.9  10.1  2.3  10.2  -0.1  100.0 | 81.3  18.7  3.5  16.1  2.7  97.3 | 80.3  19.7  4.6  19.1  0.7  99.3 | 76.2  23.8  5.8  22.1  1.7  98.3 | 74.1  25.9  6.9  32  -6.1  100.0 |

^a^ Dead cells (%) = 100% - % of cell viability

^b^ Percentage of glycerol in immunotoxins

^c^ Percentage of dead cells from glycerol treated cells from TABLE 7.1

^d^ Normalised dead cells (%) = a - c

^e^ Normalised cell viability (%) = 100% - d

^f^ Glycerol content that affect the cell viability

**MTT ASSAY ABSORBANCE READINGS OF GLYCEROL TREATED NHDF CELLS**

| Percentage (%) | | | | | | | | |
| --- | --- | --- | --- | --- | --- | --- | --- | --- |
|  | Blank | 0.0 | 1.0 | 2.5 | 5.0 | 7.0 | 10.0 | 15.0 |
| *1^st^ Replicate*  Reading 1  Reading 2  Reading 3  Average  Minus blank^a^  Standard deviation  Cell viability  *2^nd^ Replicate*  Reading 1  Reading 2  Reading 3  Average  Minus blank^a^  Standard deviation  Cell viability  Average cell viability^c^  Standard deviation  Average dead cells^d^ | 0.043  0.042  0.041  0.042  0.056  0.051  0.057  0.055 | 0.287  0.290  0.300  0.292  0.250  0.007  100.0%  0.311  0.298  0.300  0.303  0.248  0.007  100.0%  100.0%  0.009  0.0% | 0.266  0.274  0.288  0.276  0.234  0.011  93.6%  0.286  0.274  0.288  0.283  0.228  0.008  91.9%  92.8%  0.009  7.2% | 0.260  0.277  0.259  0.265  0.223  0.010  89.2%  0.277  0.260  0.279  0.272  0.217  0.010  87.5%  88.4%  0.010  11.6% | 0.252  0.235  0.241  0.243  0.201  0.001  80.4%  0.241  0.255  0.253  0.250  0.195  0.008  78.6%  79.5%  0.008  20.5% | 0.210  0.224  0.208  0.214  0.172  0.009  68.8%  0.221  0.224  0.218  0.221  0.166  0.003  66.9%  67.9%  0.007  32.1% | 0.101  0.097  0.094  0.097  0.055  0.004  22.0%  0.091  0.097  0.098  0.095  0.041  0.004  16.5%  19.3%  0.003  80.7% | 0.052  0.049  0.055  0.052  0.010  0.003  4.0%  0.062  0.069  0.055  0.062  0.007  0.007  2.8%  3.4%  0.007  96.6% |

^a^ Minus blank is average value subtracted with average of blank. Standard deviation is standard error calculate from the three readings. Percentage of cell viability is calculated based on Equation 3.6.

^c^ Average cell viability (%) is average cell viability (%) of replicate 1 and replicate 2

^d^ Average dead cells (%) = 100% - average cell viability (%)^c^

**MTT ASSAY ABSORBANCE READINGS OF MHALT-1 TREATED NHDF CELLS**

|  | Blank | 0 | 5 | 10 | 15 | 20 | 25 |
| --- | --- | --- | --- | --- | --- | --- | --- |
| *1^st^ Replicate*  Reading 1  Reading 2  Reading 3  Average  Minus blank^a^  Standard deviation  Cell viability  *2^nd^ Replicate*  Reading 1  Reading 2  Reading 3  Average  Minus blank^a^  Standard deviation  Cell viability  Average cell viability^c^  Standard deviation | 0.084  0.083  0.085  0.084  0.151  0.105  0.102  0.119 | 0.472  0.357  0.341  0.390  0.306  0.071  100.0%  0.933  0.897  0.775  0.868  0.749  0.083  100.0%  100.0%  0.271 | 0.349  0.367  0.343  0.353  0.269  0.012  87.9%  0.607  0.599  0.747  0.651  0.532  0.083  71.0%  79.4%  0.172 | 0.380  0.363  0.345  0.363  0.279  0.018  91.1%  0.731  1.768  0.844  1.114  0.995  0.569  132.8%  112.0%  0.547 | 0.359  0.362  0.321  0.347  0.263  0.023  86.1%  0.843  0.723  0.765  0.777  0.658  0.061  87.8%  86.9%  0.239 | 0.392  0.375  0.332  0.366  0.282  0.031  92.3%  0.664  0.695  0.816  0.725  0.606  0.080  80.9%  86.6%  0.204 | 0.434  0.368  0.377  0.393  0.309  0.036  100.0%  0.669  0.768  0.822  0.753  0.634  0.078  84.6%  92.8%  0.204 |

^a^ Minus blank is average value subtracted with average of blank. Standard deviation is standard error calculate from the three readings.

Percentage of cell viability is calculated based on Equation 3.6.

^c^ Average cell viability (%) is average cell viability (%) of replicate 1 and replicate 2

**MTT ASSAY ABSORBANCE READINGS OF ANTI-MKRAS G12V-34 TREATED NHDF CELLS**

|  | Blank | 0 | 5 | 10 | 15 | 20 | 25 | 30 |
| --- | --- | --- | --- | --- | --- | --- | --- | --- |
| *1^st^ Replicate*  Reading 1  Reading 2  Reading 3  Average  Minus blank^a^  Standard deviation  Cell viability  *2^nd^ Replicate*  Reading 1  Reading 2  Reading 3  Average  Minus blank^a^  Standard deviation  Cell viability  Average cell viability^c^  Standard deviation | 0.039  0.041  0.040  0.040  0.048  0.045  0.040  0.044 | 0.194  0.243  0.237  0.225  0.185  0.027  100.0%  0.214  0.217  0.250  0.227  0.183  0.020  100.0%  100.0%  0.021 | 0.210  0.202  0.248  0.220  0.180  0.025  97.5%  0.177  0.249  0.231  0.219  0.175  0.038  95.6%  96.5%  0.028 | 0.198  0.214  0.189  0.200  0.160  0.013  86.8%  0.227  0.242  0.205  0.225  0.180  0.019  98.7%  92.8%  0.020 | 0.219  0.246  0.236  0.234  0.194  0.014  104.9%  0.289  0.209  0.252  0.250  0.206  0.040  112.6%  108.7%  0.028 | 0.201  0.233  0.226  0.220  0.180  0.017  97.5%  0.256  0.238  0.201  0.232  0.187  0.028  102.6%  100.0%  0.022 | 0.236  0.214  0.247  0.232  0.192  0.017  104.2%  0.191  0.215  0.274  0.227  0.182  0.043  99.8%  102.0%  0.029 | 0.217  0.254  0.207  0.226  0.186  0.025  100.7%  0.247  0.178  0.226  0.217  0.173  0.035  94.5%  97.6%  0.028 |

^a^ Minus blank is average value subtracted with average of blank. Standard deviation is standard error calculate from the three readings.

Percentage of cell viability is calculated based on Equation 3.6.

^c^ Average cell viability (%) is average cell viability (%) of replicate 1 and replicate 2

**MTT ASSAY ABSORBANCE READINGS OF HCT 116 TREATED CELLS**

**MTT ASSAY ABSORBANCE READINGS OF SCFV-MHALT-1 TREATED HCT 116 CELLS**

| Concentration (μg/mL) | | | | | | | | | |  |
| --- | --- | --- | --- | --- | --- | --- | --- | --- | --- | --- |
|  | Blank | 0 | 5 | 10 | 15 | 20 | 25 | 30 | DMSO | Camptothecin |
| *1^st^ Replicate*  Reading 1  Reading 2  Reading 3  Average  Minus blank^a^  Standard deviation  Cell viability  *2^nd^ Replicate*  Reading 1  Reading 2  Reading 3  Average  Minus blank^a^  Standard deviation  Cell viability  Average cell viability^c^  Standard deviation  Glycerol (%) | 0.078  0.081  0.076  0.078  0.067  0.061  0.066  0.065 | 1.753  1.633  1.720  1.702  1.624  0.062  100.0%  1.353  1.339  1.321  1.338  1.273  0.016  100.0%  100.0%  0.204  0.0 | 1.340  1.412  1.266  1.339  1.261  0.073  77.6%  1.141  1.112  1.138  1.130  1.066  0.016  83.4%  80.5%  0.124  2.5 | 0.734  0.917  0.880  0.844  0.766  0.097  47.2%  0.632  0.625  0.638  0.632  0.567  0.007  44.5%  45.9%  0.131  5.0 | 0.477  0.458  0.435  0.457  0.379  0.021  23.3%  0.367  0.357  0.335  0.353  0.288  0.016  22.6%  23.0%  0.059  7.6 | 0.261  0.291  0.266  0.273  0.195  0.016  12.1%  0.161  0.194  0.166  0.174  0.109  0.018  8.56%  10.3%  0.056  10.0^b^ | 0.153  0.151  0.170  0.158  0.080  0.010  4.9%  0.153  0.151  0.170  0.158  0.093  0.011  7.3%  6.1%  0.009  12.7^b^ | 0.107  0.113  0.113  0.111  0.033  0.003  2.0%  0.091  0.093  0.091  0.092  0.027  0.001  2.1%  2.1%  0.011  15.3^b^ | 0.145  0.134  0.192  0.157  0.079  0.031  4.9%  0.098  0.096  0.092  0.095  0.031  0.003  2.4%  3.7%  0.039  - | 0.844  0.754  0.789  0.796  0.718  0.045  44.2%  0.723  0.598  0.745  0.689  0.624  0.079  49.0%  46.6%  0.082  - |

^a^ Minus blank is average value subtracted with average of blank. Standard deviation is standard error calculate from the three readings.

Percentage of cell viability is calculated based on Equation 3.6.

^b^ Glycerol content that affect the cell viability.

^c^ Average cell viability (%) is average cell viability (%) of replicate 1 and replicate 2

**NORMALISED ABSORBANCE READINGS OF SCFV-MHALT-1 IMMUNOTOXIN TREATED HCT 116 CELLS**

| Concentration (μg/mL) | | | | | | | |
| --- | --- | --- | --- | --- | --- | --- | --- |
|  | 0 | 5 | 10 | 15 | 20 | 25 | 30 |
| Cell viability (%)  Dead cells (%)^a^  Glycerol (%)^b^  Dead cells (%)^c^  Normalised dead cells (%)^d^  Normalised cell viability (%)^e^ | 100.0  0.0  0.0  0.0  0.0  100.0 | 80.5  19.5  2.5  19.3  0.2  99.8 | 45.9  54.1  5.0  19.2  34.9  65.1 | 23.0  77.0  7.6  32.0  45.0  55.0 | 10.3  89.7  10.0  79.6  10.1  89.9 | 6.1  93.9  12.7  85.1  8.8  91.2 | 2.1  97.9  15.3  97.8  0.1  99.9 |

^a^ Dead cells (%) = 100% - % of cell viability

^b^ Percentage of glycerol in immunotoxins

^c^ Percentage of dead cells from glycerol treated cells from TABLE 7.6

^d^ Normalised dead cells (%) = a - c

^e^ Normalised cell viability (%) = 100% - d

^f^ Glycerol content that affect the cell viability

**MTT ASSAY ABSORBANCE READINGS OF MHALT-1-SCFV TREATED HCT 116 CELLS**

| Concentration (μg/mL) | | | | | | | | | |
| --- | --- | --- | --- | --- | --- | --- | --- | --- | --- |
|  | Blank | 0 | 5 | 10 | 15 | 20 | 25 | 30 | DMSO |
| *1^st^ Replicate*  Reading 1  Reading 2  Reading 3  Average  Minus blank^a^  Standard deviation  Cell viability  *2^nd^ Replicate*  Reading 1  Reading 2  Reading 3  Average  Minus blank^a^  Standard deviation  Cell viability  Average cell viability^c^  Standard deviation  Glycerol (%) | 0.078  0.079  0.081  0.079  0.067  0.061  0.066  0.065 | 1.791  1.796  1.789  1.792  1.713  0.004  100.0%  1.361  1.344  1.346  1.350  1.286  0.009  100.0%  100.0%  0.242  0.0 | 1.512  1.479  1.571  1.521  1.442  0.047  84.2%  1.114  1.108  1.111  1.111  1.046  0.003  81.3%  82.8%  0.226  1.2 | 1.211  1.212  1.201  1.208  1.129  0.006  66.0%  0.987  0.982  0.978  0.982  0.918  0.005  71.2%  68.6%  0.124  2.3 | 1.182  1.197  1.197  1.192  1.113  0.009  65.0%  0.961  0.961  0.978  0.967  0.902  0.010  70.1%  67.6%  0.124  3.5 | 0.602  0.984  0.961  0.849  0.770  0.214  45.0%  0.602  0.689  0.665  0.652  0.587  0.045  45.6%  45.3%  0.176  4.6 | 0.620  0.669  0.692  0.660  0.581  0.037  33.9%  0.488  0.453  0.492  0.478  0.413  0.021  32.1%  33.0%  0.104  5.8 | 0.623  0.563  0.576  0.587  0.508  0.032  29.7%  0.401  0.399  0.389  0.396  0.332  0.006  26.8%  27.8%  0.107  6.9 | 0.145  0.134  0.192  0.157  0.079  0.031  4.6%  0.098  0.096  0.092  0.095  0.031  0.003  2.3%  3.5%  0.039  - |

^a^ Minus blank is average value of each concentration subtracted with average of blank. Standard deviation is standard error calculate from the three readings. Percentage of cell viability is calculated based on Equation 3.6.

^c^ Average cell viability (%) is average cell viability value of replicate 1 and replicate 2

**NORMALISED ABSORBANCE READINGS OF MHALT-1-SCFV IMMUNOTOXIN TREATED HCT 116 CELLS**

| Concentration (μg/mL) | | | | | | | |
| --- | --- | --- | --- | --- | --- | --- | --- |
|  | 0 | 5 | 10 | 15 | 20 | 25 | 30 |
| Cell viability (%)  Dead cells (%)^a^  Glycerol (%)^b^  Dead cells (%)^c^  Normalised dead cells (%)^d^  Normalised cell viability (%)^e^ | 100.0  0.0  0.0  0.0  0.0  100.0 | 83.0  17.0  1.2  1.8  15.2  84.8 | 68.6  31.4  2.3  18.5  12.9  87.1 | 67.6  32.4  3.5  19.2  13.2  86.8 | 45.3  54.7  4.6  18.9  35.8  64.2 | 33.0  67.0  5.8  21.7  45.3  54.7 | 27.8  72.2  6.9  27.7  44.5  55.5 |

^a^ Dead cells (%) = 100% - % of cell viability

^b^ Percentage of glycerol in immunotoxins

^c^ Percentage of dead cells from glycerol treated cells from TABLE 7.6

^d^ Normalised dead cells (%) = a - c

^e^ Normalised cell viability (%) = 100% - d

^f^ Glycerol content that affect the cell viability

**MTT ASSAY ABSORBANCE READINGS OF GLYCEROL TREATED HCT 116 CELLS**

| Percentage (%) | | | | | | | | |
| --- | --- | --- | --- | --- | --- | --- | --- | --- |
|  | Blank | 0.0 | 1.0 | 2.5 | 5.0 | 7.0 | 10.0 | 15.0 |
| *1^st^ Replicate*  Reading 1  Reading 2  Reading 3  Average  Minus blank^a^  Standard deviation  Cell viability  *2^nd^ Replicate*  Reading 1  Reading 2  Reading 3  Average  Minus blank^a^  Standard deviation  Cell viability  Average cell viability^c^  Standard deviation  Average dead cells^d^ | 0.079  0.078  0.081  0.079  0.081  0.079  0.078  0.079 | 1.759  1.733  1.721  1.738  1.659  0.020  100.0%  1.359  1.333  1.341  1.344  1.265  0.013  100.0%  100.0%  0.216  0.0% | 1.832  1.712  1.629  1.724  1.645  0.102  99.2%  1.332  1.318  1.323  1.324  1.245  0.007  98.4%  98.8%  0.228  1.2% | 1.379  1.412  1.363  1.385  1.306  0.025  78.7%  1.123  1.119  1.131  1.124  1.045  0.006  82.6%  80.7%  0.144  19.3% | 1.369  1.396  1.436  1.400  1.321  0.034  79.6%  1.122  1.116  1.111  1.116  1.037  0.006  82.0%  80.8%  0.157  19.2% | 1.151  1.270  1.328  1.250  1.171  0.090  70.6%  0.987  0.981  0.989  0.986  0.906  0.004  71.6%  71.1%  0.155  28.9% | 0.405  0.422  0.481  0.436  0.357  0.040  21.5%  0.311  0.322  0.338  0.324  0.244  0.014  19.3%  20.4%  0.067  79.6% | 0.158  0.125  0.132  0.138  0.059  0.017  3.6%  0.091  0.113  0.121  0.108  0.029  0.016  2.3%  3.0%  0.022  97.0% |

^a^ Minus blank is average value subtracted with average of blank. Standard deviation is standard error calculate from the three readings. Percentage of cell viability is calculated based on Equation 3.6.

^c^ Average cell viability (%) is average cell viability (%) of replicate 1 and replicate 2

^d^ Average dead cells (%) = 100% - average cell viability (%)^c^

**MTT ASSAY ABSORBANCE READINGS OF MHALT-1 TREATED HCT 116 CELLS**

|  | Blank | 0 | 5 | 10 | 15 | 20 | 25 |
| --- | --- | --- | --- | --- | --- | --- | --- |
| *1^st^ Replicate*  Reading 1  Reading 2  Reading 3  Average  Minus blank^a^  Standard deviation  Cell viability  *2^nd^ Replicate*  Reading 1  Reading 2  Reading 3  Average  Minus blank^a^  Standard deviation  Cell viability  Average cell viability^c^  Standard deviation | 0.265  0.204  0.293  0.254  0.148  0.139  0.169  0.152 | 0.848  0.876  0.825  0.850  0.596  0.026  100.0%  0.897  0.976  1.180  1.018  0.866  0.146  100.0%  100.0%  0.131 | 0.722  0.887  0.821  0.810  0.556  0.083  93.3%  0.941  0.836  0.820  0.866  0.714  0.066  82.4%  87.9%  0.074 | 0.884  0.795  0.730  0.803  0.549  0.077  92.2%  0.983  0.943  0.966  0.964  0.812  0.020  93.8%  93.0%  0.102 | 0.852  0.803  0.842  0.832  0.578  0.026  97.1%  1.049  0.886  0.841  0.925  0.773  0.109  89.3%  93.2%  0.088 | 0.902  0.781  0.728  0.804  0.550  0.089  92.3%  1.058  0.849  0.995  0.967  0.815  0.107  94.2%  93.2%  0.126 | 0.889  0.798  0.799  0.829  0.575  0.052  96.5%  0.997  1.045  0.886  0.976  0.824  0.082  95.2%  95.8%  0.101 |

^a^ Minus blank is average value subtracted with average of blank. Standard deviation is standard error calculate from the three readings.

Percentage of cell viability is calculated based on Equation 3.6.

^c^ Average cell viability (%) is average cell viability (%) of replicate 1 and replicate 2

**MTT ASSAY ABSORBANCE READINGS OF ANTI-MKRAS G12V-34 TREATED HCT 116 CELLS**

|  | Blank | 0 | 5 | 10 | 15 | 20 | 25 | 30 |
| --- | --- | --- | --- | --- | --- | --- | --- | --- |
| *1^st^ Replicate*  Reading 1  Reading 2  Reading 3  Average  Minus blank^a^  Standard deviation  Cell viability  *2^nd^ Replicate*  Reading 1  Reading 2  Reading 3  Average  Minus blank^a^  Standard deviation  Cell viability  Average cell viability^c^  Standard deviation | 0.051  0.054  0.049  0.051  0.049  0.054  0.057  0.053 | 0.797  0.629  0.650  0.692  0.641  0.092  100.0%  0.792  0.726  0.745  0.754  0.701  0.034  100.0%  100.0%  0.071 | 0.605  0.442  0.633  0.560  0.509  0.103  79.4%  0.638  0.660  0.636  0.645  0.591  0.013  84.4%  81.9%  0.080 | 0.624  0.641  0.576  0.614  0.562  0.034  87.8%  0.613  0.622  0.633  0.623  0.569  0.010  81.2%  84.5%  0.023 | 0.768  0.558  0.780  0.702  0.651  0.125  101.6%  0.637  0.567  0.618  0.607  0.554  0.036  79.0%  90.3%  0.097 | 0.596  0.574  0.614  0.595  0.543  0.020  84.8%  0.633  0.520  0.677  0.610  0.556  0.081  79.4%  82.1%  0.053 | 0.565  0.695  0.666  0.642  0.591  0.068  92.2%  0.613  0.538  0.603  0.585  0.531  0.041  75.8%  84.0%  0.059 | 0.635  0.583  0.700  0.639  0.588  0.059  91.8%  0.678  0.659  0.644  0.660  0.607  0.017  86.6%  89.2%  0.040 |

^a^ Minus blank is average value subtracted with average of blank. Standard deviation is standard error calculate from the three readings.

Percentage of cell viability is calculated based on Equation 3.6.

^c^ Average cell viability (%) is average cell viability (%) of replicate 1 and replicate 2

**MTT ASSAY ABSORBANCE READINGS OF SW-480 TREATED CELLS**

**MTT ASSAY ABSORBANCE READINGS OF SCFV-MHALT-1 TREATED SW-480 CELLS**

| Concentration (μg/mL) | | | | | | | | | |  |
| --- | --- | --- | --- | --- | --- | --- | --- | --- | --- | --- |
|  | Blank | 0 | 5 | 10 | 15 | 20 | 25 | 30 | DMSO | Camptothecin |
| *1^st^ Replicate*  Reading 1  Reading 2  Reading 3  Average  Minus blank^a^  Standard deviation  Cell viability  *2^nd^ Replicate*  Reading 1  Reading 2  Reading 3  Average  Minus blank^a^  Standard deviation  Cell viability  Average cell viability^c^  Standard deviation  Glycerol (%) | 0.081  0.084  0.083  0.083  0.091  0.089  0.093  0.091 | 1.656  1.612  1.653  1.640  1.557  0.025  100.0%  2.134  2.232  2.211  2.192  2.101  0.052  100.0%  100.0%  0.304  0.0 | 1.645  1.624  1.654  1.641  1.558  0.015  100.0%  2.089  1.998  2.008  2.032  1.941  0.050  92.4%  96.2%  0.217  2.5 | 1.220  1.236  1.284  1.247  1.164  0.033  74.8%  1.623  1.639  1.643  1.635  1.544  0.011  73.5%  74.2%  0.214  5.0 | 0.478  0.514  0.502  0.498  0.415  0.018  26.7%  0.738  0.714  0.732  0.728  0.637  0.012  30.3%  28.5%  0.127  7.6 | 0.435  0.434  0.410  0.426  0.343  0.014  22.0%  0.699  0.685  0.676  0.687  0.596  0.012  28.4%  25.2%  0.143  10.0^b^ | 0.361  0.352  0.364  0.359  0.276  0.006  17.7%  0.564  0.552  0.545  0.554  0.463  0.010  22.0%  19.9%  0.107  12.7^b^ | 0.295  0.292  0.267  0.285  0.202  0.015  13.0%  0.495  0.482  0.467  0.481  0.390  0.014  18.6%  15.8%  0.109  15.3^b^ | 0.378  0.324  0.450  0.384  0.301  0.063  19.3%  0.548  0.524  0.531  0.534  0.443  0.012  21.1%  20.2%  0.092  - | 0.886  0.819  0.854  0.853  0.770  0.034  49.5%  0.967  1.131  0.988  1.029  0.938  0.089  44.6%  47.1%  0.114 |

^a^ Minus blank is average value subtracted with average of blank. Standard deviation is standard error calculate from the three readings.

Percentage of cell viability is calculated based on Equation 3.6.

^b^ Glycerol content that affect the cell viability.

^c^ Average cell viability (%) is average cell viability (%) of replicate 1 and replicate 2

**NORMALISED ABSORBANCE READINGS OF SCFV-MHALT-1 IMMUNOTOXIN TREATED SW-480 CELLS**

| Concentration (μg/mL) | | | | | | | |
| --- | --- | --- | --- | --- | --- | --- | --- |
|  | 0 | 5 | 10 | 15 | 20 | 25 | 30 |
| Cell viability (%)  Dead cells (%)^a^  Glycerol (%)^b^  Dead cells (%)^c^  Normalised dead cells (%)^d^  Normalised cell viability (%)^e^ | 100.0  0.0  0.0  0.0  0.0  100.0 | 96.2  3.8  2.5  7.3  -3.5  103.5 | 74.2  25.8  5.0  8.9  16.9  83.1 | 28.5  71.5  7.6  13.0  58.5  41.5 | 25.2  74.8  10.0  67.8  7.0  93.0 | 19.9  80.1  12.7  75.4  4.7  95.3 | 15.8  84.2  15.3  84.0  0.2  99.8 |

^a^ Dead cells (%) = 100% - % of cell viability

^b^ Percentage of glycerol in immunotoxins

^c^ Percentage of dead cells from glycerol treated cells from TABLE 8.1

^d^ Normalised dead cells (%) = a - c

^e^ Normalised cell viability (%) = 100% - d

^f^ Glycerol content that affect the cell viability

**MTT ASSAY ABSORBANCE READINGS OF MHALT-1-SCFV TREATED SW-480 CELLS**

| Concentration (μg/mL) | | | | | | | | | |
| --- | --- | --- | --- | --- | --- | --- | --- | --- | --- |
|  | Blank | 0 | 5 | 10 | 15 | 20 | 25 | 30 | DMSO |
| *1^st^ Replicate*  Reading 1  Reading 2  Reading 3  Average  Minus blank^a^  Standard deviation  Cell viability  *2^nd^ Replicate*  Reading 1  Reading 2  Reading 3  Average  Minus blank^a^  Standard deviation  Cell viability  Average cell viability^c^  Standard deviation  Glycerol (%) | 0.082  0.083  0.082  0.082  0.091  0.089  0.093  0.091 | 1.851  1.870  1.860  1.860  1.778  0.010  100.0%  2.217  2.197  2.213  2.209  2.118  0.011  100.0%  100.0%  0.191  0.0 | 1.875  1.871  1.871  1.872  1.790  0.002  100.7%  2.189  2.211  2.198  2.199  2.108  0.012  99.5%  100.1%  0.179  1.2 | 1.752  1.645  1.692  1.796  1.696  0.054  95.4%  2.067  1.987  1.972  2.009  1.918  0.051  90.6%  93.0%  0.177  2.3 | 1.281  1.288  1.252  1.274  1.192  0.019  67.0%  1.471  1.476  1.452  1.466  1.375  0.013  64.9%  66.0%  0.107  3.5 | 0.909  0.992  0.972  0.958  0.876  0.043  49.3%  1.111  1.097  1.107  1.105  1.014  0.007  47.9%  48.6%  0.085  4.6 | 0.789  0.725  0.702  0.739  0.657  0.045  37.0%  0.989  0.973  0.981  0.981  0.890  0.008  42.0%  39.5%  0.136  5.8 | 0.542  0.505  0.533  0.527  0.445  0.019  25.0%  0.722  0.705  0.733  0.720  0.629  0.014  29.7%  27.4%  0.107  6.9 | 0.378  0.324  0.450  0.384  0.301  0.063  16.9%  0.518  0.484  0.511  0.504  0.413  0.018  19.5%  18.2%  0.078  - |

^a^ Minus blank is average value of each concentration subtracted with average of blank. Standard deviation is standard error calculate from the three readings. Percentage of cell viability is calculated based on Equation 3.6.

^c^ Average cell viability (%) is average cell viability value of replicate 1 and replicate 2

**NORMALISED ABSORBANCE READINGS OF MHALT-1-SCFV IMMUNOTOXIN TREATED SW-480 CELLS**

| Concentration (μg/mL) | | | | | | | |
| --- | --- | --- | --- | --- | --- | --- | --- |
|  | 0 | 5 | 10 | 15 | 20 | 25 | 30 |
| Cell viability (%)  Dead cells (%)^a^  Glycerol (%)^b^  Dead cells (%)^c^  Normalised dead cells (%)^d^  Normalised cell viability (%)^e^ | 100.0  0.0  0.0  0.0  0.0  100.0 | 100.1  -0.1  1.2  2.5  -2.6  102.6 | 93.0  7.0  2.3  6.9  0.1  99.9 | 66.0  34.0  3.5  8.1  26.9  74.1 | 48.6  51.4  4.6  8.6  42.8  57.2 | 39.5  60.5  5.8  9.4  51.1  48.9 | 27.4  72.6  6.9  12.1  60.5  39.5 |

^a^ Dead cells (%) = 100% - % of cell viability

^b^ Percentage of glycerol in immunotoxins

^c^ Percentage of dead cells from glycerol treated cells from TABLE 8.1

^d^ Normalised dead cells (%) = a - c

^e^ Normalised cell viability (%) = 100% - d

^f^ Glycerol content that affect the cell viability

**MTT ASSAY ABSORBANCE READINGS OF GLYCEROL TREATED SW-480 CELLS**

| Percentage (%) | | | | | | | | |
| --- | --- | --- | --- | --- | --- | --- | --- | --- |
|  | Blank | 0.0 | 1.0 | 2.5 | 5.0 | 7.0 | 10.0 | 15.0 |
| *1^st^ Replicate*  Reading 1  Reading 2  Reading 3  Average  Minus blank^a^  Standard deviation  Cell viability  *2^nd^ Replicate*  Reading 1  Reading 2  Reading 3  Average  Minus blank^a^  Standard deviation  Cell viability  Average cell viability^c^  Standard deviation  Average dead cells^d^ | 0.081  0.082  0.081  0.081  0.091  0.089  0.093  0.091 | 1.597  1.622  1.535  1.585  1.504  0.045  100.0%  2.221  2.177  2.195  2.198  2.107  0.022  100.0%  100.0%  0.337  0.0% | 1.529  1.546  1.538  1.538  1.457  0.009  96.9%  2.179  2.186  2.171  2.179  2.088  0.008  99.1%  98.0%  0.351  2.0% | 1.496  1.422  1.400  1.439  1.358  0.050  90.3%  2.091  2.099  2.095  2.095  2.004  0.004  95.1%  92.7%  0.361  7.3% | 1.453  1.409  1.436  1.433  1.352  0.022  89.9%  2.046  2.037  2.025  2.036  1.945  0.011  92.3%  91.1%  0.331  8.9% | 1.363  1.368  1.373  1.368  1.287  0.005  85.6%  1.989  1.968  1.972  1.976  1.885  0.011  89.5%  87.6%  0.333  12.4% | 0.508  0.555  0.536  0.533  0.452  0.024  30.1%  0.821  0.813  0.805  0.813  0.722  0.008  34.3%  32.2%  0.154  67.8% | 0.279  0.288  0.311  0.293  0.212  0.017  14.1%  0.529  0.489  0.512  0.510  0.419  0.020  19.9%  17.0%  0.120  83.0% |

^a^ Minus blank is average value subtracted with average of blank. Standard deviation is standard error calculate from the three readings. Percentage of cell viability is calculated based on Equation 3.6.

^c^ Average cell viability (%) is average cell viability (%) of replicate 1 and replicate 2

^d^ Average dead cells (%) = 100% - average cell viability (%)

**MTT ASSAY ABSORBANCE READINGS OF MHALT-1 TREATED SW-480 CELLS**

|  | Blank | 0 | 5 | 10 | 15 | 20 | 25 |
| --- | --- | --- | --- | --- | --- | --- | --- |
| *1^st^ Replicate*  Reading 1  Reading 2  Reading 3  Average  Minus blank^a^  Standard deviation  Cell viability  *2^nd^ Replicate*  Reading 1  Reading 2  Reading 3  Average  Minus blank^a^  Standard deviation  Cell viability  Average cell viability^c^  Standard deviation | 0.168  0.123  0.136  0.142  0.250  0.143  0.162  0.185 | 1.055  0.915  1.282  1.084  0.942  0.185  100.0%  1.161  0.856  0.876  0.964  0.779  0.171  100.0%  100.0%  0.131 | 1.043  0.880  1.073  0.999  0.856  0.104  90.9%  0.972  0.959  0.813  0.915  0.730  0.088  93.6%  92.3%  0.098 | 1.302  0.785  1.204  1.097  0.955  0.275  101.4%  0.980  1.224  0.830  1.011  0.826  0.199  106.0%  103.7%  0.220 | 0.980  0.856  1.125  0.987  0.845  0.135  89.7%  1.100  1.145  0.907  1.051  0.866  0.126  111.1%  100.4%  0.122 | 0.885  0.972  1.023  0.960  0.818  0.070  86.8%  1.107  1.054  0.855  1.005  0.820  0.133  105.3%  96.0%  0.098 | 0.969  0.950  0.653  0.857  0.715  0.177  75.9%  0.996  1.136  1.051  1.061  0.876  0.071  112.4%  94.2%  0.164 |

^a^ Minus blank is average value subtracted with average of blank. Standard deviation is standard error calculate from the three readings.

Percentage of cell viability is calculated based on Equation 3.6.

^c^ Average cell viability (%) is average cell viability (%) of replicate 1 and replicate 2

**MTT ASSAY ABSORBANCE READINGS OF ANTI-MKRAS G12V-34 TREATED SW-480 CELLS**

|  | Blank | 0 | 5 | 10 | 15 | 20 | 25 | 30 |
| --- | --- | --- | --- | --- | --- | --- | --- | --- |
| *1^st^ Replicate*  Reading 1  Reading 2  Reading 3  Average  Minus blank^a^  Standard deviation  Cell viability  *2^nd^ Replicate*  Reading 1  Reading 2  Reading 3  Average  Minus blank^a^  Standard deviation  Cell viability  Average cell viability^c^  Standard deviation | 0.043  0.041  0.042  0.042  0.038  0.041  0.047  0.042 | 0.463  0.499  0.506  0.489  0.447  0.023  100.0%  0.491  0.509  0.576  0.525  0.483  0.045  100.0%  100.0%  0.037 | 0.440  0.403  0.461  0.435  0.393  0.029  87.8%  0.513  0.450  0.438  0.467  0.425  0.040  87.9%  87.9%  0.036 | 0.694  0.462  0.512  0.556  0.514  0.122  114.9%  0.486  0.458  0.448  0.464  0.422  0.020  87.3%  101.1%  0.093 | 0.439  0.457  0.476  0.457  0.415  0.019  92.8%  0.465  0.455  0.427  0.449  0.407  0.020  84.2%  88.5%  0.018 | 0.450  0.590  0.485  0.508  0.466  0.073  104.2%  0.505  0.455  0.495  0.485  0.443  0.026  91.7%  98.0%  0.051 | 0.482  0.486  0.486  0.485  0.443  0.002  99.0%  0.432  0.435  0.443  0.437  0.395  0.006  81.7%  90.3%  0.027 | 0.452  0.534  0.509  0.498  0.456  0.042  102.0%  0.468  0.478  0.497  0.481  0.439  0.015  90.8%  96.4%  0.030 |

^a^ Minus blank is average value subtracted with average of blank. Standard deviation is standard error calculate from the three readings.

Percentage of cell viability is calculated based on Equation 3.6.

^c^ Average cell viability (%) is average cell viability (%) of replicate 1 and replicate 2
