# Supplementary material for: Development of a single-chain fragment variable fused-mutant HALT-1 recombinant immunotoxin against G12V mutated KRAS colorectal cancer cells
Source: PeerJ. 2021 Apr 15;9:e11063. doi: 10.7717/peerj.11063 (PMC8053384; doi:10.7717/peerj.11063)
Supplement: Supplemental Information 4 [file peerj-09-11063-s004.docx]

**CELL-BASED ELISA ASSAY ABSORBANCE READINGS OF SCFV-MHALT-1 IMMUNOTOXIN TREATED CELLS**

**CELL-BASED ELISA ASSAY ABSORBANCE READINGS OF SCFV-MHALT-1 IMMUNOTOXIN TREATED NHDF CELLS**

|  |  | Concentration (μg/mL) | | |
| --- | --- | --- | --- | --- |
|  | **Non-coated** | **0^b^** | **10** | **20** |
| *Replicate 1*  Reading 1  Reading 2  Average  Standard deviation  Relative mean^a^  *Replicate 2*  Reading 1  Reading 2  Average  Standard deviation  Relative mean^a^  Average relative mean  Standard deviation | 0.061  0.059  0.060  0.001  -  0.060  0.056  0.058  0.003  - | 0.423  0.375  0.399  0.034  0.000  0.397  0.355  0.376  0.030  0.000  0.000  0.029 | 0.463  0.479  0.471  0.011  0.012  0.426  0.443  0.435  0.012  0.001  0.006  0.023 | 0.524  0.511  0.518  0.009  0.059  0.497  0.501  0.499  0.003  0.065  0.062  0.012 |

^a^ Relative mean value is average value subtracted with average of non-coated and blank^b^. Standard deviation is standard error calculated from the two readings.

^b^ Blank with cell-coated wells without immunotoxin (untreated cell) were used to identify background signals

**CELL-BASED ELISA ASSAY ABSORBANCE READINGS OF SCFV-MHALT-1 IMMUNOTOXIN TREATED HCT 116 CELLS**

|  |  | Concentration (μg/mL) | | |
| --- | --- | --- | --- | --- |
|  | **Non-coated** | **0^b^** | **10** | **20** |
| *Replicate 1*  Reading 1  Reading 2  Average  Standard deviation  Relative mean^a^  *Replicate 2*  Reading 1  Reading 2  Average  Standard deviation  Relative mean^a^  Average relative mean  Standard deviation | 0.061  0.059  0.060  0.001  -  0.060  0.056  0.058  0.003  - | 0.321  0.354  0.338  0.023  0.000  0.453  0.448  0.451  0.004  0.000  0.000  0.067 | 0.757  0.732  0.745  0.018  0.347  0.787  0.761  0.774  0.018  0.266  0.306  0.023 | 0.603  0.578  0.591  0.018  0.193  0.677  0.656  0.667  0.015  0.158  0.176  0.046 |

^a^ Relative mean value is average value subtracted with average of non-coated and blank^b^. Standard deviation is standard error calculated from the two readings.

^b^ Blank with cell-coated wells without immunotoxin (untreated cell) were used to identify background signals

**CELL-BASED ELISA ASSAY ABSORBANCE READINGS OF SCFV-MHALT-1 IMMUNOTOXIN TREATED SW-480 CELLS**

|  |  | Concentration (μg/mL) | | |
| --- | --- | --- | --- | --- |
|  | **Non-coated** | **0^b^** | **10** | **20** |
| *Replicate 1*  Reading 1  Reading 2  Average  Standard deviation  Relative mean^a^  *Replicate 2*  Reading 1  Reading 2  Average  Standard deviation  Relative mean^a^  Average relative mean  Standard deviation | 0.061  0.059  0.060  0.001  -  0.060  0.056  0.058  0.003  - | 0.523  0.506  0.515  0.012  0.000  0.638  0.642  0.640  0.003  0.000  0.000  0.073 | 0.758  0.781  0.770  0.016  0.195  0.793  0.799  0.796  0.004  0.098  0.147  0.018 | 0.719  0.700  0.710  0.013  0.135  0.889  0.896  0.893  0.005  0.195  0.165  0.106 |

^a^ Relative mean value is average value subtracted with average of non-coated and blank^b^. Standard deviation is standard error calculated from the two readings.

^b^ Blank with cell-coated wells without immunotoxin (untreated cell) were used to identify background signals

**CELL-BASED ELISA ASSAY ABSORBANCE READINGS OF MHALT-1-SCFV IMMUNOTOXIN TREATED CELLS**

**CELL-BASED ELISA ASSAY ABSORBANCE READINGS OF MHALT-1-SCFV MMUNOTOXIN TREATED NHDF CELLS**

|  |  | Concentration (μg/mL) | | |
| --- | --- | --- | --- | --- |
|  | **Non-coated** | **0^b^** | **10** | **20** |
| *Replicate 1*  Reading 1  Reading 2  Average  Standard deviation  Relative mean^a^  *Replicate 2*  Reading 1  Reading 2  Average  Standard deviation  Relative mean^a^  Average relative mean  Standard deviation | 0.074  0.073  0.074  0.001  -  0.071  0.069  0.070  0.002  - | 0.498  0.485  0.492  0.009  0.000  0.412  0.354  0.383  0.041  0.000  0.000  0.067 | 0.567  0.573  0.570  0.004  0.005  0.459  0.448  0.454  0.008  0.001  0.003  0.067 | 0.591  0.601  0.596  0.007  0.031  0.487  0.488  0.488  0.001  0.035  0.033  0.063 |

^a^ Relative mean value is average value subtracted with average of non-coated and blank^b^. Standard deviation is standard error calculated from the two readings.

^b^ Blank with cell-coated wells without immunotoxin (untreated cell) were used to identify background signals

**CELL-BASED ELISA ASSAY ABSORBANCE READINGS OF MHALT-1-SCFV IMMUNOTOXIN TREATED HCT 116 CELLS**

|  |  | Concentration (μg/mL) | | |
| --- | --- | --- | --- | --- |
|  | **Non-coated** | **0^b^** | **10** | **20** |
| *Replicate 1*  Reading 1  Reading 2  Average  Standard deviation  Relative mean^a^  *Replicate 2*  Reading 1  Reading 2  Average  Standard deviation  Relative mean^a^  Average relative mean  Standard deviation | 0.074  0.073  0.074  0.001  -  0.071  0.069  0.070  0.001  - | 0.381  0.400  0.391  0.013  0.000  0.321  0.320  0.321  0.001  0.000  0.000  0.041 | 0.583  0.568  0.576  0.011  0.112  0.483  0.492  0.488  0.006  0.097  0.104  0.051 | 0.721  0.732  0.727  0.008  0.263  0.671  0.684  0.678  0.009  0.287  0.275  0.029 |

^a^ Relative mean value is average value subtracted with average of non-coated and blank^b^. Standard deviation is standard error calculated from the two readings.

^b^ Blank with cell-coated wells without immunotoxin (untreated cell) were used to identify background signals

**CELL-BASED ELISA ASSAY ABSORBANCE READINGS OF MHALT-1-SCFV MMUNOTOXIN TREATED SW-480 CELLS**

|  |  | Concentration (μg/mL) | | |
| --- | --- | --- | --- | --- |
|  | **Non-coated** | **0^b^** | **10** | **20** |
| *Replicate 1*  Reading 1  Reading 2  Average  Standard deviation  Relative mean^a^  *Replicate 2*  Reading 1  Reading 2  Average  Standard deviation  Relative mean^a^  Average relative mean  Standard deviation | 0.074  0.073  0.074  0.001  -  0.071  0.069  0.070  0.001  - | 0.389  0.412  0.401  0.016  0.000  0.331  0.330  0.331  0.001  0.000  0.000  0.041 | 0.432  0.444  0.438  0.008  0.000  0.423  0.400  0.412  0.016  0.011  0.000  0.019 | 0.886  0.905  0.896  0.013  0.422  0.772  0.791  0.782  0.013  0.381  0.401  0.067 |

^a^ Relative mean value is average value subtracted with average of non-coated and blank^b^. Standard deviation is standard error calculated from the two readings.

^b^ Blank with cell-coated wells without immunotoxin (untreated cell) were used to identify background signals
