# Supplementary material for: Development of a single-chain fragment variable fused-mutant HALT-1 recombinant immunotoxin against G12V mutated KRAS colorectal cancer cells
Source: PeerJ. 2021 Apr 15;9:e11063. doi: 10.7717/peerj.11063 (PMC8053384; doi:10.7717/peerj.11063)
Supplement: Supplemental Information 5 — Chromatogram of pCANTAB5E:G12V-34, pCANTAB5E:G12V-50, pCANTAB5E:G13D-5, pCANTAB5E:G13D-18 DNA sequencing results. [file peerj-09-11063-s005.rtf]

pCANTAB5E:G12V-34Name: BankIt2368183 Seq1 Accession number: MT799824 TNNGNNNATTNCACGTGAAAAATTATTATTCGCAATTCCTTTAGTTGTTCCTTTCTATGCGGCCCAGCCGGCCATGGCCCAGGTCCAACTGCAGCAGCCTGGGGCTGAGCTGGTGAGGCCTGGAGCTTCAGTGAAGCTGTCCTGCAAGACTTCTGGCTACTCCTTCACCAGCTACTGGATGAACTGGGTAAAACAGAGGCCTGGACAGGGTCTGGAATGGATTGGATACATTAATCCTAGCACTGGTTATACTGAGTACAATCAGAAGTTCAAGGACAAGGCCACATTGACTGCAGACAAATCCTCCAGCACAGCCTACATGCAACTGAGCAGCCTGACATCTGAGGACTCTGCAGTCTATTACTGTGCAAGAAGGGACTATAGGTACTTCGATGTCTGGGGCGCAGGGACCACGGTCACCGTCTCCTCAGGTGGAGGCGGTTCAGGCGGAGGTGGCTCTGGCGGTGGCGGATCGGACATTGTGATGACCCAGTCTCCAGCACTCATGTCTGCAGCTCTAGGGGAACGGGTCACCATGACCTGCAGTGCCAGCTCAAGTGTAAGTTACATGCACTGGTACCAGCAGAAGTCAGGCACCTCCCCCAAAAGATGGATTTATGACACATCCAAACTGGCTTCTGGAGTCCCTGCTCGCTTCAGTGGCAGTGGGTCTGGGACCTCTTACTCTCTCACAATCAGCAGCATGGAGGCTGAAGATGCTGCCACTTATTACTGCCAGCAGTGGAGTAGTAACCCATTCACGTTCGGCTCGGGGACAAAGTTGGAAATAAAACGGGCGGCCGCAGGTGCGCCGGTGCCGTATCCGGATCCGCTGGAACCGCGTGCCGCATAGACTGTTGAAAGTTGTTTAGCAAAACCTCATACAGAAAATTCATTTACTAACGTCTGGAAAGACGACAAAACTTTAGATCGTTACGCTAACTATGAGGGCTGTCTGTGGAATGCTACAGGCGTTGTGGTTTGTACTGGTGACGAAACTCAGTGTTACGGTACATGGGTTCCTATTGGGCTTGCTATCCCTGAAAATGAGGGTGGTGGCTCTGAGGGTGGCGGTTCTGAGGGTGGCGGTTCTGAAGGTGGCGGTACTAAACCTCCTGAGTACGGTGATACACCTATTCCGGGCTATACTTATATCAACCCTCTCGACGGCACTTATCCGCCTGGTACTGAGCAAAACNCCGCTAATCCTAATCCTTNNNTTGAGGAATCTCAGCCTCTTAATACTTTCATGTTTCAAAATAATAGGTTCCAAATAGGCAGGGTGCATTAACTGTTTANCGGGCACTGTTACTCAAGGCNCTGACCCCGTTAAACTTATTACCAGTACCTCCTGATCATCAAAGCCATGTATGACCTTACTGAACGGTAAAT
