# Supplementary figures and images for: Development of a single-chain fragment variable fused-mutant HALT-1 recombinant immunotoxin against G12V mutated KRAS colorectal cancer cells
Source: PeerJ. 2021 Apr 15;9:e11063. doi: 10.7717/peerj.11063 (PMC8053384; doi:10.7717/peerj.11063)

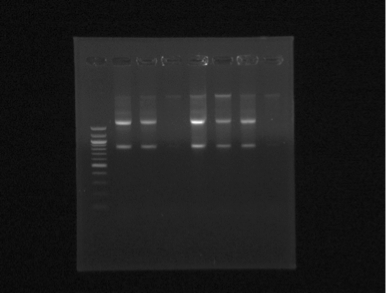

Supplement: Supplemental Information 6 — Figures 1(a), 1(b), 1(c), 3, 6(a), 6(b), 6(c), 7(a), 7(b). [file peerj-09-11063-s006.zip › Figure 1 (a).png]

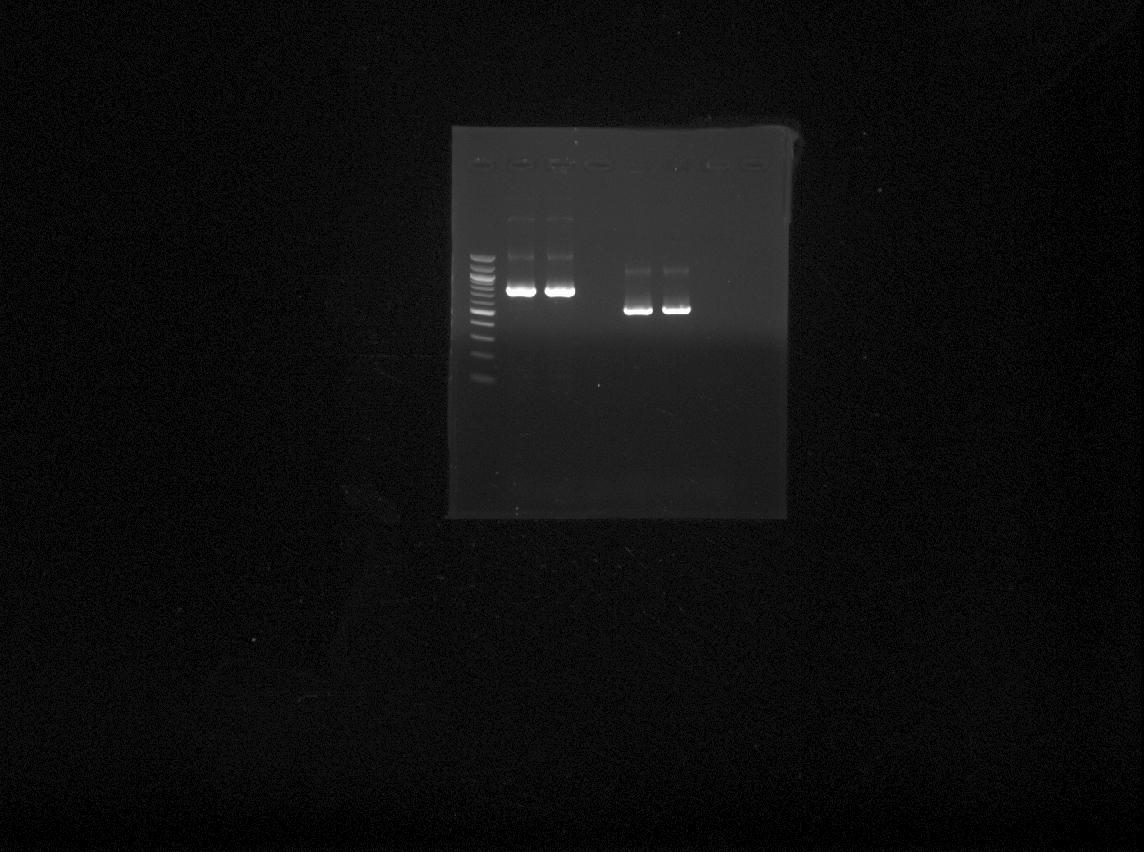

Supplement: Supplemental Information 6 — Figures 1(a), 1(b), 1(c), 3, 6(a), 6(b), 6(c), 7(a), 7(b). [file peerj-09-11063-s006.zip › Figure 6 (a).jpg]

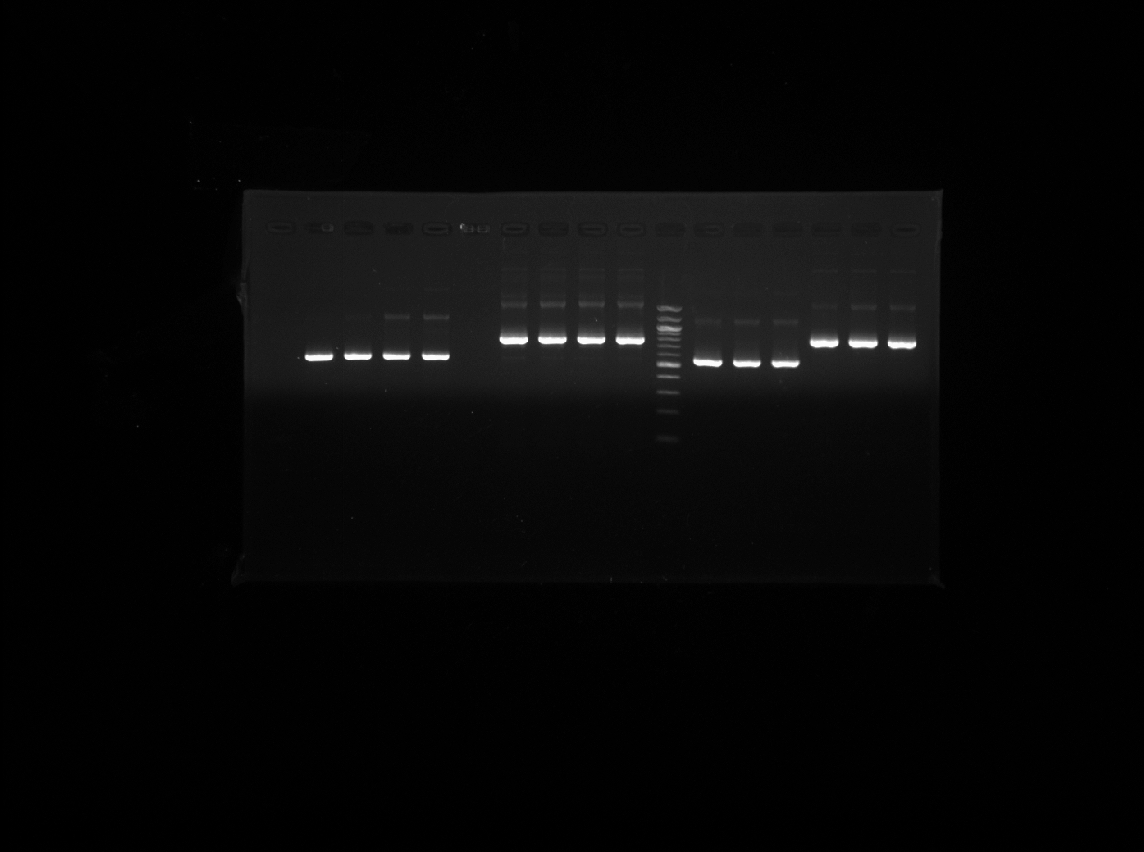

Supplement: Supplemental Information 6 — Figures 1(a), 1(b), 1(c), 3, 6(a), 6(b), 6(c), 7(a), 7(b). [file peerj-09-11063-s006.zip › Figure 6 (b).jpg]

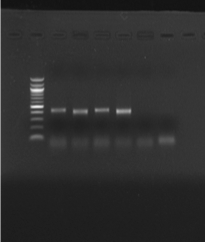

Supplement: Supplemental Information 6 — Figures 1(a), 1(b), 1(c), 3, 6(a), 6(b), 6(c), 7(a), 7(b). [file peerj-09-11063-s006.zip › Figure 1 (b).png]

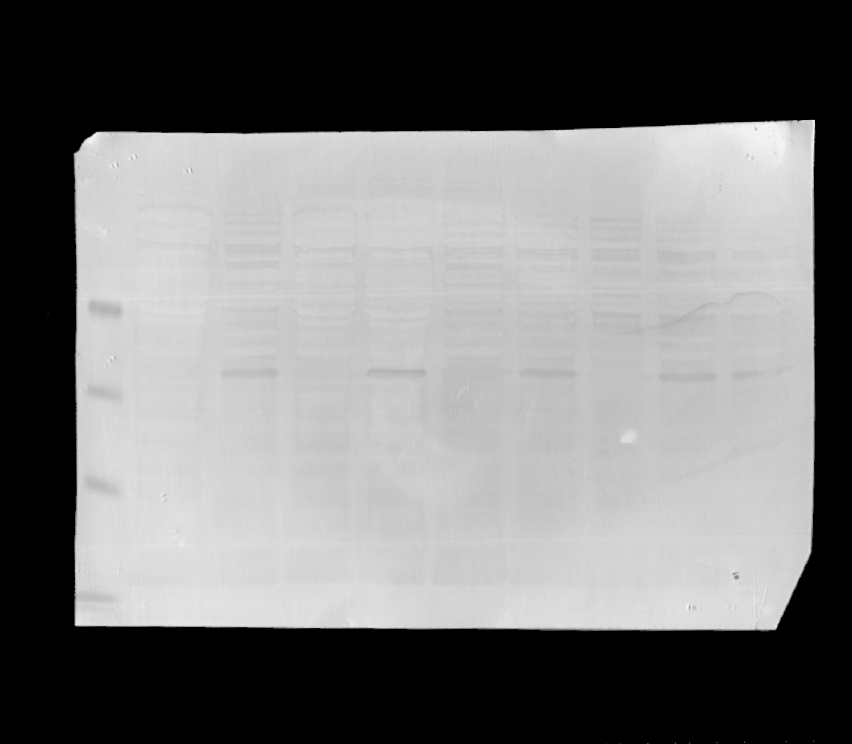

Supplement: Supplemental Information 6 — Figures 1(a), 1(b), 1(c), 3, 6(a), 6(b), 6(c), 7(a), 7(b). [file peerj-09-11063-s006.zip › Figure 3.jpg]

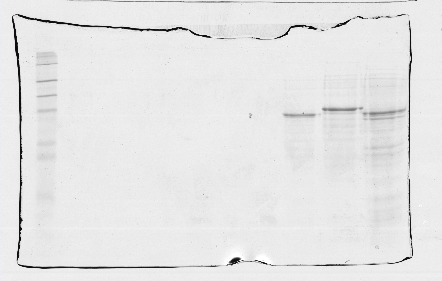

Supplement: Supplemental Information 6 — Figures 1(a), 1(b), 1(c), 3, 6(a), 6(b), 6(c), 7(a), 7(b). [file peerj-09-11063-s006.zip › Figure 7 (b).jpg]

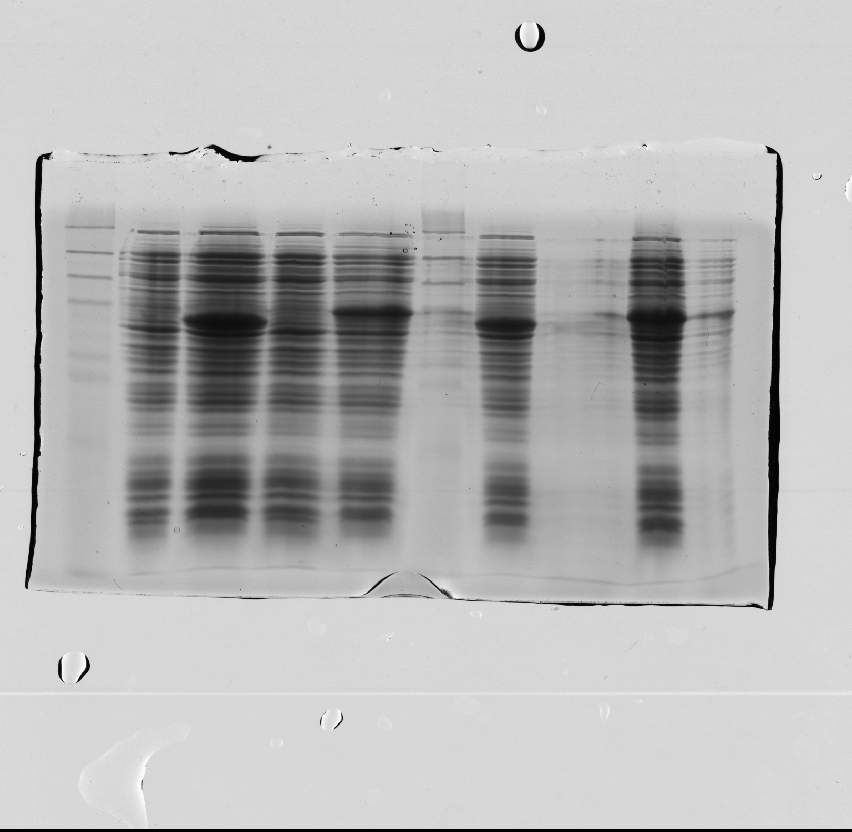

Supplement: Supplemental Information 6 — Figures 1(a), 1(b), 1(c), 3, 6(a), 6(b), 6(c), 7(a), 7(b). [file peerj-09-11063-s006.zip › Figure 7(a).jpg]

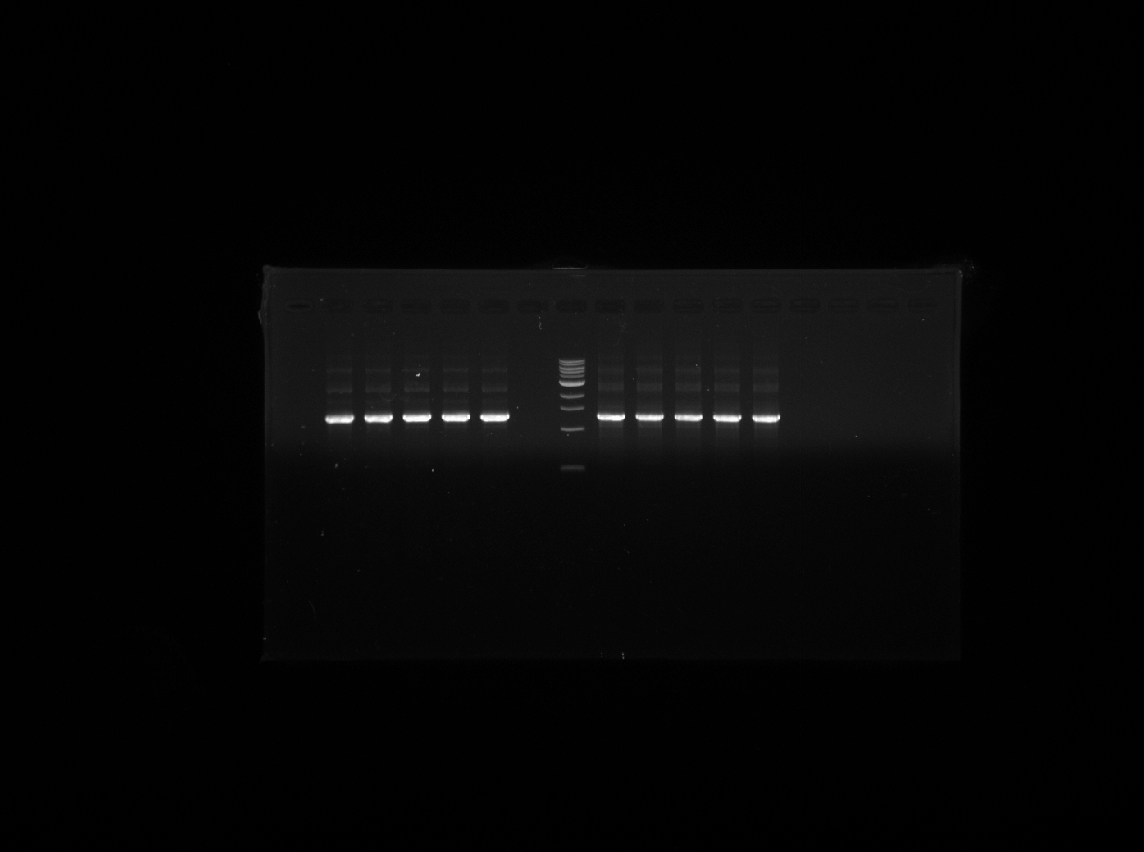

Supplement: Supplemental Information 6 — Figures 1(a), 1(b), 1(c), 3, 6(a), 6(b), 6(c), 7(a), 7(b). [file peerj-09-11063-s006.zip › Figure 6 (c).jpg]

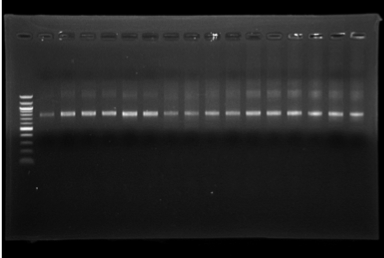

Supplement: Supplemental Information 6 — Figures 1(a), 1(b), 1(c), 3, 6(a), 6(b), 6(c), 7(a), 7(b). [file peerj-09-11063-s006.zip › Figure 1 (c).png]
